# A new *Apicomplexa*-specific protein kinase family : multiple members in *Plasmodium falciparum*, all with an export signature

**DOI:** 10.1186/1471-2164-6-30

**Published:** 2005-03-07

**Authors:** Achim G Schneider, Odile Mercereau-Puijalon

**Affiliations:** 1 Unité d'Immunologie Moléculaire des Parasites, CNRS URA 2581, Institut Pasteur, Paris, France

## Abstract

**Background:**

Malaria caused by protozoan parasites of the genus *Plasmodium spp*. is a major health burden in tropical countries. The development of new control tools, including vaccines and drugs, is urgently needed. The availability of genome sequences from several malaria parasite species provides a basis on which to identify new potential intervention targets. Database mining for orthologs to the *Plasmodium falciparum *trophozoite protein R45, a vaccine candidate, led us identify a new gene family.

**Results:**

Orthologs to the *P. falciparum *trophozoite protein R45 were detected exclusively in protozoan parasites of the phylum *Apicomplexa*, including several *Plasmodium spp*., *Toxoplasma gondii and Cryptosporidium parvum*. All family members are hybrid genes with a conserved C-terminal protein kinase domain of a novel type, recently called FIKK kinase, associated with a non conserved N-terminal region without any known functional signature. While a single copy gene was detected in most species, considerable gene expansion was observed in *P. falciparum *and its closest phylogenic relative *P. reichenowi*, with 20 and six copies, respectively, each with a distinct N-terminal domain. Based on full length protein sequence, pairs of orthologs were observed in closely related species, such as *P. berghei *and *P.y. yoelii*, *P. vivax *and *P. knowlesi*, or *P. reichenowi *and *P. falciparum*. All 20 *P. falciparum *paralogs possess a canonical *Plasmodium *export element downstream of a signal / anchor sequence required for exportation outside the parasitophorous vacuole. This is consistent with the reported association of the trophozoite protein R45, the only paralog characterised to date, with the infected red blood cell membrane. Interestingly, most genes are located in the subtelomeric region of chromosomes, in association with other multigene families contributing to the remodelling of the infected red blood cell membrane, in particular the ring erythrocyte surface antigen multigene family.

**Conclusion:**

This Apicomplexan-specific gene family was called R45-FIKK kinase. The family hallmark is a kinase domain with unusual characteristics, raising the possibility of designing drug or vaccine strategies targeting this domain. The characteristics of the *P. falciparum *family suggest a role in remodelling the infected cell and as such possibly contribute to the particular virulence of this species.

## Background

*Plasmodium spp*. are protozoan parasites (phylum *Apicomplexa*, class *Sporozoa*, order *Haemosporida*) with a complex life cycle, including alternating development phases in mosquito and vertebrate hosts. More than a hundred *Plasmodium *species have been described, of which four infect humans and cause malaria. The genome sequences of the most pathogenic human species, *P. falciparum *[1], and of *P. yoelii yoelii*, a murine malaria species [2], were published in 2002. Sequencing of additional *Plasmodium *species is underway. Recently, the genome of *Cryptosporidium parvum *another Apicomplexan parasite has been published [3], while the genome of *Toxoplasma gondii *is being sequenced. The body of information available offers new opportunities in malaria research, and will hopefully accelerate rational design of novel intervention strategies, a pressing need in view of the rapidly deteriorating efficiency of existing control tools.

In recent years, our laboratory has explored the vaccine potential of the trophozoite protein R45, a *P. falciparum *antigen exported by the parasite to the infected red blood cell membrane [4]. The protein contains a large central domain with 90 copies of a HK/MSDH/SN consensus hexapeptide, encoded by the gene annotated as PFD1175w in the *P. falciparum *genome [1]. We have shown that a recombinant antigen called R23, which contains 11 such repeats is the target of antibodies promoting phagocytosis of parasitized red blood cells [5] and induces strong protective immunity against lethal infection in *Saimiri sciureus *squirrel monkeys [6-8]. Furthermore, the response to R23 repeats is associated with protection against clinical malaria in humans [9]. The PFD1175w gene sequence is well conserved among all isolates of *P. falciparum *studied so far. In order to obtain information on the organisational and expression constraints of the antigen, we carried out a comparative genomic approach. This showed that the N-terminal region and the central hexapeptide repeats were *P. falciparum*-specific and unique to PFD1175w. In contrast, multiple paralogs of the C-terminal region were identified in *P. falciparum *and a single ortholog in most other *Plasmodium *species as well as in some distantly related Apicomplexan species. This C-terminal region is predicted to be a protein kinase domain [4], with high divergence from all other known kinase domains. We describe here some characteristics by this novel gene family and of this novel kinase domain, and discuss the possible relevance of species-specific gene amplification.

## Results

### Identification of R45 paralogs in *P. falciparum*

**Figure 1:**
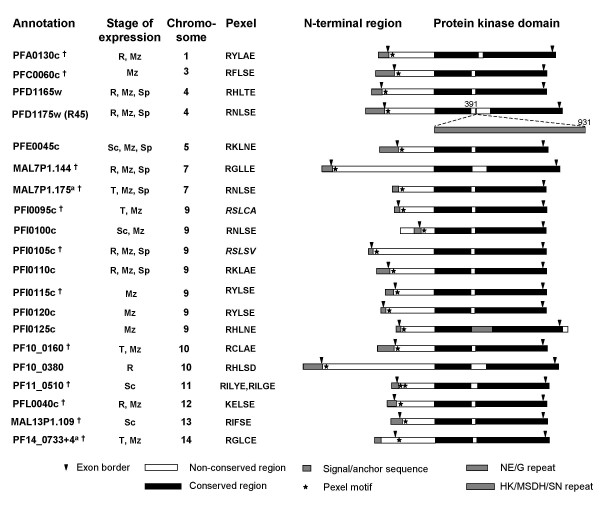
Schematic representation of the R45-FIKK kinase genes in the *P. falciparum *3D7 genome including salient protein features as deduced from PlasmoDB (as per release 4.3) and mRNA expression profiling as published by Bozdech et al [16] and Le Roch et al [17]. The 20 genes of the family are scattered over 11 chromosomes, as indicated. Stage of expression refers to the stages in the life cycle where the mRNA has been consistently detected and / or is maximal (R, T, Sc, Mz and Sp refer to ring stage, trophozoite, schizont, merozoite and sporozoite, respectively). The kinase domain is depicted as a black bar. The polymorphic region within the kinase domain is shown in white. Exon borders are indicated with a downwards arrow head. The location of the pentameric Plasmodium Export element (Pexel) is marked with an asterisk. The Pexel sequence is indicated for each deduced protein. It is indicated in italics when it does not entirely match with the consensus (R,K)XLX(D,Q,E). ^† ^genes re-annotated in this work (see Materials and Methods).

The R45 trophozoite protein (PFD1175w) has a predicted three-exon structure, with a small (201 bp) exon 1, a large (3387 bp) exon 2 followed by a short (78 bp) exon 3. Gene structure and exon / intron boundaries were experimentally confirmed by cDNA sequencing (data not shown). Based on homology with PFD1175w, a gene family with 20 paralogs was identified in the genome of the 3D7 clone of *P. falciparum*. Data mining indicated that all 20 paralogs are transcribed at a stage or another of the life cycle, and for many members at multiple stages (Figure 1) [16,17]. Twelve genes were re-annotated to fit with the gene structure and amino acid sequence of the other paralogs (see Materials and Methods). All genes have a three exon structure with a large exon 2 flanked by two small exons, except Mal7P1.175, where the small exon 1 appears to be missing (or fused to exon 2) (Figure 1). Two paralogs (Mal7P1.175 and PF14_0733+4) have an internal stop codon, reflecting pseudogenes or need for read through translation [18].

The hallmark of the family is a conserved C-terminal domain showing 38 – 64 % identity among the paralogs, with its terminal 24 – 41 residues encoded by the small exon 3 [exon 2 in Mal7P1.175] (Figure 1). This conserved domain has characteristic features of protein kinases, but can not be assigned to any known kinase family. The presence of all amino acids necessary for phosphotransfer except the canonical ATP fixation motif GxGxxG in all 20 paralogs [see Additional file 1] suggests a novel protein kinase type. This is consistent with the conclusions of Ward et al, who independently identified this protein kinase family, that was called FIKK-kinase, using a whole genome search for protein kinases in *P. falciparum *[19]. Two members contain a repeat region inserted between subdomains III and IV consisting of 90 copies of the H K S/N D N/H/S N hexapeptide in PFD1175w and of 32 copies of the two amino acid motif NE/G in PFI0125. The other family members contain a stretch of up to 53 non-conserved and non-repeated amino acids in this region, as illustrated in Figure 1.

Contrasting with the conserved C-terminal kinase domain, the N-terminal region is unique to each paralog, with less than 20% identity and substantial size variation (ranging from 124 residues for PFI0100c to 471 residues for PF10_0380). Homology and motif searches for each unique N-terminal region did not identify any known Pfam domain or a significant homology with any other *P. falciparum *protein. However, all twenty paralogs contain a stretch of hydrophobic amino acids within the first 70 amino acids, corresponding to a predicted trans-membrane or signal sequence for 14 members according to PlasmoDB. Analysis of the re-annotated paralogs using an improved signal peptide prediction algorithm [10] and manual curation identified a likely signal / anchor sequence in all but one paralogs (Figure 1). For PFI0100c, there was no signal / anchor sequence probability but there was a trans-membrane prediction. We considered here as a possible signal / anchor sequence that might have escaped detection by the algorithms used. The secretion motif RxSRILAExxx identified recently [20] was present in six paralogs (PFD1165w, PFI0105c, PF10_0160, PFL0040c, MAL13P1.109, PF14_0733+4). Interestingly however, the shorter *Plasmodium *export element (Pexel) identified independently by Marti et al [21] was detected in all paralogs downstream of the signal / anchor sequence (Figure 1) [see Additional file 2]. The consensus (R,K) × (L,I) × (E,Q,D) pentamer was observed in 18 paralogs. A non-canonical amino acid in the fifth position was found in PFI0095c and PFI00105c (A and V, respectively). The Pexel motif is located 16–24 amino acids downstream of the start of the second exon, except in Mal7P1.175, where it is 11 residues downstream of the signal sequence. Pf11_510 has two putative Pexel motifs located at positions 20–24 and 44–48.

### R45-FIKK kinase orthologs in other Plasmodium species and in other Apicomplexans

Orthologs were identified in most *Plasmodium *genome sequences available to date. A single copy gene was observed in the human species *P. vivax *(PvR45), the simian species *P. knowlesi *(PkR45), the murine species *P.y. yoelii *(PyR45) and *P. berghei *(PbR45), and the avian species *P. gallinacaeum *(PgR45). In contrast, six paralogs were identified in *P. reichenowi *(PrR45-1-6). No ortholog was identified in the murine species *P. chabaudi*, possibly reflecting the low coverage (2×) of the available genome sequence.

**Figure 2:**
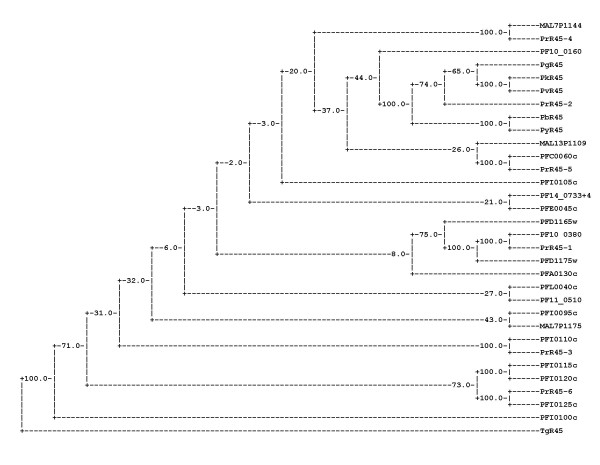
Phylogenetic distance of 32 R45-FIKK kinases as inferred from their C-terminal FIKK kinase domain. A consensus tree was built with the *T. gondii *sequence as outgroup. Numbers indicate bootstrap values, with 100 corresponding to a perfect cluster.

**Figure 3:**
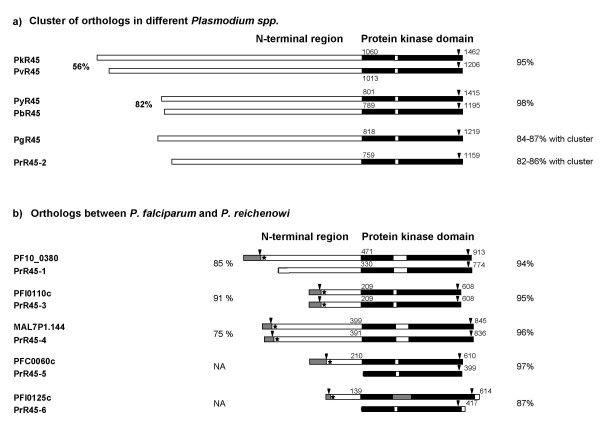
Schematic representation of R45-FIKK kinase genes in several *Plasmodium *species. a) Clustering of the R45-FIKK kinase genes into a single ortholog group. b) Orthology between *P. falciparum *and *P. reichenowi*. The degree of identity of the N-terminal domain and of the kinase domain between the orthologous pairs is indicated on the left and right hand side, respectively. NA = not applicable. Symbols are as in Figure 1.

The deduced protein sequence alignment showed the FIKK-protein kinase domain to be well conserved within the genus [see Additional file 1]. None of the non *P. falciparum *orthologs identified here contain any repeat region. Based on their kinase domain, the R45-FIKK kinase proteins from six *Plasmodium *species, namely PvR45, PkR45, PyR45, PbR45, PgR45 and PrR45-2, clustered perfectly in a phylogenetic tree with 82 – 98 % identity (Figure 2), suggesting that they derive from the same common ancestor. Intriguingly, no *P. falciparum *R45-like protein was observed in this cluster. The best match, Pf10_0160, showed a non-significant bootstrap of 44. The six R45-like orthologs of the core cluster are quite distinct from the *P. falciparum *R45-FIKK genes with regard to their gene structure and N-terminal region (Figure 3a) [see Additional file 3]. They have a two exon structure. The short, highly conserved second exon is the ortholog of the conserved third *P. falciparum *exon. All genes lack an ortholog of the first *P. falciparum *exon and hence encode a protein without a signal sequence and a Pexel motif. In addition, the N-terminal region is much larger than for any *P. falciparum *paralog, ranging from 1060 residues in PkR45 to 759 residues in PrR45-2. Interestingly, analysis of the N-terminal region identified pairs of orthologs, with 56 % identity between PvR45 and PkR45, and 82 % identity between PyR45 and PbR45 (Figure 3a). These pairs form two quite separate homology groups, with less than 20 % overall inter-group identity. Despite of the low overall conservation, stretches of conserved amino acids are present within the 200 downstream residues of the N-terminal region [see Additional file 3]. These motifs are not observed in any *P. falciparum *ortholog [see Additional file 2].

*P. reichenowi*, like *P. falciparum*, has multiple R45-FIKK kinase paralogs. As indicated above, PrR45-2, clusters with the orthologs from the single copy species and does not have an ortholog in the 3D7 *P. falciparum *genome. Interestingly however, the five other *P. reichenowi *paralogs could each be attributed to a specific *P. falciparum *R45-FIKK kinase paralog (Figure 3b), forming orthologous pairs as follows: PrR45-1 – PF10_380, PrR45-3 – PFI0110, PrR45-4 – Mal7P1.144, PrR45-5 – PFC0060, PrR45-6 – PFI0125. For those *P. reichenowi *paralogs were enough sequence data was available, there was remarkable conservation of both the kinase domain (87 – 97 % identity with the respective *P. falciparum *ortholog) and the N-terminal region (75–91 % idem). Interestingly, the two *P. reichenowi *orthologs with full length sequence available, PrR45-3 and PrR45-4, share the 3 exon structure with their *P. falciparum *orthologs, have an exon 1-encoded signal sequence and a conserved Pexel sequence close to the start of exon 2. Progress in the *P. reichenowi *genome should indicate whether or not the other orthologs present the same structure so far observed only in *P. falciparum *and *P. reichenowi*.

A single copy R45-like ortholog was identified in *T. gondii *(TgR45) as well as in *C. parvum *(CpR45), two parasite species that belong to a distinct class, namely *Coccidea*, within the phylum *Apicomplexa*. Their deduced amino acid sequence is quite divergent from the *Plasmodium spp*. family members (Additional file 1 and 2). The protein kinase domain of CpR45 and TgR45 shows 41 – 42 % and 31 – 33 % identity, respectively, with the cluster of orthologs. The lower value for TgR45 is explained by insertions of stretches of amino acids between some subdomains. The gene structure of TgR45 and CpR45 differs considerably from their orthologs in the various *Plasmodium spp*. TgR45 is encoded by a minimum of seven exons, with possible additional upstream exons. While the seventh exon corresponds to the third exon of the *P. falciparum *paralogs, the remaining kinase domain is encoded by six exons. A signal sequence is predicted within the first 21 amino acids of TgR45, but not followed by a Pexel motif. The significance of this finding is unclear, because the actual start of the TgR45coding region is not yet identified. In contrast, in line with the general paucity of introns in the *C. parvum *genome, CpR45 is encoded by a single exon [3]. The C-terminal amino acids encoded by a specific exon in all the other orthologs are present and well conserved in CpR45.

### Chromosome localisation in *P. falciparum*

**Figure 4:**
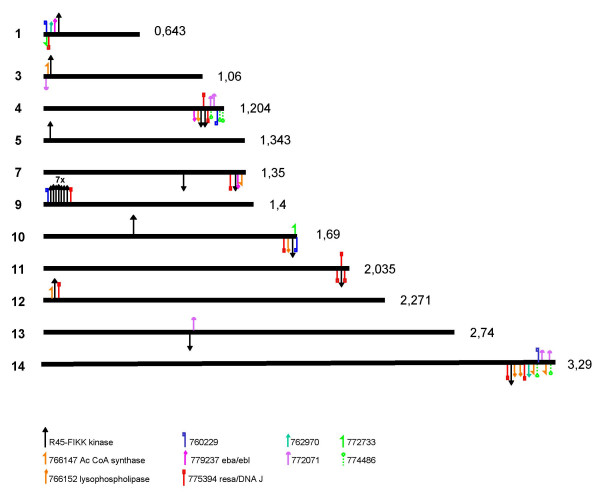
Chromosomal localisation of R45-FIKK kinase genes and of some associated multigene families in the *P. falciparum *3D7 genome, as deduced from the maps published by Gardner et al [1] and the version 4.3 genome annotation from PlasmoDB. The genes transcribed leftwards (annotated "c" in the 3D7 genome) are placed above the chromosome. The genes transcribed rightwards (annotated "w" in the 3D7 genome) are placed beneath the chromosome. Total chromosome length as deduced from PlasmoDB (as per December 2004) is given in megabases on the right. Note that for chromosomes whose sequence is still incomplete, the actual chromosome size is slightly larger. The subtelomeric regions are not drawn to scale.

The 20 *P. falciparum *paralogs are distributed over 11 chromosomes. Interestingly, 17 of 20 R45-FIKK kinase paralogs are located within 150 kb from their telomer, all with the sense of transcription directed towards the telomer (Figure 4). Seven of these paralogs are single copy on their respective chromosome. A cluster of seven head to tail parologs is located on chromosome 9, and two paralogs are separated by a single gene on chromosome 4. Chromosomes 7 and 10 have two paralogs, one in the sub-telomeric region and one in the central region. Chromosome 13 has a single centrally located gene.

Interestingly, the subtelomeric R45-FIKK kinase paralogs are consistently associated with paralogs from other multigene families with a predominant subtelomeric localisation. The most frequent association (15 of 17 R45-FIKK kinase subtelomeric paralogs) is with the ortholog group named 775394, which contains proteins with a DNA J domain, in particular, the ring erythrocyte surface antigens (*resa *1–3). There seems to be a close association between both families as in most cases the 775394 paralog is the closest or the next closest gene to a R45-FIKK paralog. For instance, PFD1165w and PFD1175w, which are located 80 kb from the chromosome 4 telomer, are each flanked in 3' by a 775394 paralog. Likewise, PF11_0510 is located between the *resa2 *and *resa3 *paralogs on chromosome 11. PFA0130c is located further apart from *resa1*, but still is closely linked (less than 20 kb downstream of *resa1*). Approximately half of the 27 paralogs within the 775394 group are localised close to a R45-FIKK kinase gene. Additional subtelomeric gene families in the close vicinity of a R45-FIKK paralog include the 76615 (lysophospholipase), 779237 (erythrocyte binding antigens *eba *and *ebl*), 766147 (fatty acid CoA synthase) as well as several multigene families coding for hypothetical transmembrane proteins (see Figure 4). There are several examples of specific tandem arrangements such as *resa*, R45-like, *eba *(see Figure 4), suggesting an ancient higher order organisation.

*C. parvum *is the only other Apicomplexan for which chromosome maps have been established [3]. CpR45 (annotated cgd5_4390) is located approx. 35 kb from the putative telomer on chromosome 5 [3], but does not seem to be associated with any specific subtelomeric family. In *P.y. yoelii*, the R45-FIKK ortholog is not present in a subtelomeric or telomeric contig [2], but the exact chromosomal localisation is unfortunately neither known for this species nor for other *Plasmodium *species, since at this stage of genome annotation and assembly, chromosome maps are quite incomplete. A partial synteny map has been established for some regions of the *P.y. yoelii *and *P. falciparum *chromosomes. Interestingly enough, many R45-FIKK kinase paralogs lie at the boundary of the subtelomeric region where both species diverge substantially and as such correspond to breakpoint in synteny. We looked systematically for *P.y. yoelii *orthologs in the vicinity of the various R45-FIKK kinase paralogs. We observed break of synteny close to all telomeric paralogs, confirming observations by other authors [2]. In particular the synteny reported for the *P.y. yoelii *chromosome 5 and *P. falciparum *4 stops close to the localisation of PFD1165w [2]. We also observed breaks of synteny close to internal R45-FIKK paralogs, in particular downstream of the internal gene MAL7P1.144, and upstream and downstream of PF10_0160. We could not explore the vicinity of MAL13P1.109, which is still uncertain. Interestingly, the *P. vivax *contig that harbours PvR45 also contains a break in synteny *with P. falciparum*.

## Discussion

The family identified here on the basis of similarity with the non repeated regions from PFD1175w groups genes coding for a well conserved C-terminal protein kinase domain. Recently, Ward et al identified the same family using a whole genome mining for protein kinases. Based on a conserved motif from sub-domain II, they named this family "FIKK-like kinase" [19]. Since the kinase activity has not been formally demonstrated, and since the only paralog characterised in some detail so far is R45 (PFD1175w) [4-9], we prefer to name it the R45-FIKK kinase family.

The R45-FIKK family was observed only in protozoan parasites of the phylum *Apicomplexa*. A single copy gene was detected in most species except in *P. falciparum *and its closest relative *P. reichenowi*, where multiple copies were observed. As current genome coverage is ≥ 10× for *C. parvum *[3], *P. vivax *and *T. gondii*, 5× for *P. yoelii *[2] and *P. knowlesi*, the conclusion of a low (single) copy number is a fair statement. Due to incomplete coverage of the *P. berghei*, *P. gallinacaeum *and *P. chabaudi *genome sequence (2–3×), a definitive conclusion on the actual copy number in these species cannot be drawn, but a large copy number is highly unlikely. Conversely, copy number in *P. reichenowi *may be underestimated. Indeed, six copies have been detected in the available *P. reichenowi *sequence, currently only at 1.5× coverage. Additional copies might be uncovered upon completion of genome sequence.

The kinase domain is well conserved in all family members identified. It has many features of a catalytic protein kinase domain. The limited homology with well characterised kinase domains from other species precludes predicting a possible kinase type and phosphorylation target. This is in line with the observation that protozoan parasites such as *Plasmodium spp*., *T. gondii *and *C. parvum *have a quite different kinase repertoire as compared to yeast and metazoa [19,22]. The fact that all residues essential for phosphotransfer are present in each kinase domain of the R45-FIKK kinase family suggests that these proteins function as protein kinases. These observations call for experimental investigation of the potential enzymatic activity of the R45-like proteins, the uniqueness of which could represent an attractive drug or vaccine target.

The N-terminal region is much less conserved than the kinase domain but interestingly, clear ortholog pairs with very good conservation were identified. Clustering of the two rodent species *P. berghei *and *P. yoelii*, of the human *P. vivax *and simian *P. knowlesi *and of *P. falciparum *and *P. reichenowi *is in line with the phylogeny inferred from rRNA and CSP gene sequences [23,24]. The N-terminal domain of the R45-FIKK kinases may thus serve as a sensitive marker for genetic distance within the *Plasmodium *genus.

The conservation of the C-terminal FIKK-kinase domain and the restricted conservation of the N-terminal domain points to a hybrid gene, with individual components evolving at distinct rates. Evolution of the kinase domain may be constrained in order to retain enzymatic activity. In contrast, the high divergence of N-terminal regions among R45-like protein orthologs in distant species might reflect different (species- or host-specific) interaction partners and / or their possible implication in distinct biological processes or distinct stages of the life cycle. The sequence variation between the various *P. falciparum *and *P. reichenowi *paralogs is another sign of the rapid evolution of this gene family. The observation that none of the 20 *P. falciparum *R45-FIKK kinase genes clustered with the "core" group of orthologs from the other *Plasmodium *species may be related to this rapid divergence rate. Alternatively, it may merely reflect absence of this family member from the 3D7 genome, which presents several large sub-telomeric deletions and may lack the "core" ortholog. The ongoing sequencing of additional *P. falciparum *lines will clarify this issue.

PFD1175w, the trophozoite protein R45 that led us initiate this analysis, turned out to be a quite unusual family member in that it contains a large repeat region absent from all other R45-like proteins. PFD1175w clusters with the orthologous pair PrR45-1 – PF10_0380, suggesting that it originates from duplication of the latter after speciation, and has subsequently acquired the repeat region. This is the first evidence of such a process in malaria parasites. PFI0125c, which also contains a specific repeat region, clusters with a different *P. reichenowi *paralog, namely PrR45-6. Both PrR45-1 and PrR45-6 are devoid of repeats. Thus, in both cases, acquisition of repeats was *P. falciparum*-specific and posterior to the *P. reichenowi */*P. falciparum *branching. Interestingly, the repeats are anchored within a gene region that shows substantial intra-family sequence polymorphism. This gene family thus represents a unique opportunity to analyse the genesis and possible evolution of these intriguing low complexity sequences in *P. falciparum *parasites. Indeed, we have observed considerable variation in the copy number of the PFD1175w hexapeptide repeats in field isolates (Schneider et al, unpublished).

*P. falciparum *and *P. reichenowi *present an expanded set of R45-FIKK kinase genes, which furthermore have unique structural features. The genes have a three exon structure and encode a protein with canonical export signatures (signal / anchor sequence preceding a Pexel motif). This was observed in all twenty *P. falciparum *paralogs and in the *P. reichenowi *paralogs for which full length coding sequence could be retrieved. No such export signature was observed in any of the other orthologs from the family. Presence of a signal sequence associated with a downstream Pexel motif is necessary and sufficient for exportation of the protein beyond the parasitophorous vacuole [21]. This suggests that all 20 *P. falciparum *paralogs have the capacity to be exported beyond the parasitophorous vacuole membrane. The available mRNA expression profiling indicates that all 20 paralogs are transcribed by blood stages and that seven are also transcribed in sporozoites [16,17]. Evidence for protein expression was obtained for some paralogs by mass spectroscopy at the trophozoite and / or sporozoite stage [25]. Absence of protein detection must be interpreted with caution, because it may reflect low abundance of the protein in question at the developmental stage investigated. PFD1175w, the only paralog characterised to date is exported to the red blood cell membrane at the early trophozoite stage [5,6]. Immunisation of *Saimiri *monkeys with a recombinant protein derived from its central repeat region resulted in either complete protection or in selection of parasite mutants that no longer expressed red blood cell surface variant antigens [6]. It is tempting to speculate that the R45 trophozoite antigen is involved in some sort of signalling cascade, and by extrapolation that the other family members are implicated in signalling from the extracellular / extravacuolar milieu as well.

Interestingly, most *P. falciparum *R45-FIKK kinase paralogs are located in the subtelomeric regions that harbour numerous multigene families that code for proteins destined to be exported to the host cellular membrane or host cytoplasm [1,20,21]. The telomeric and subtelomeric regions of *Plasmodium spp*. harbour several multigene families that code for products involved in antigenic variation [1,2]. Remarkably enough, these multigene families differ markedly in *P. falciparum *as compared to *P.y. yoelii *[2]. Nine telomeric / subtelomeric multigene families have been identified in *P.y. yoelii*. Four of them, namely the *yir *or *vir *or the *pystb-d *families have no ortholog in the 3D7 genome, while the others are either expanded or contracted as compared to *P. falciparum *[2]. Similarly, the telomeric *var*, *rif *and *stevor P. falciparum *multigene families do not have orthologs in *P.y. yoelii *[2] or *P. vivax *[26]. Our observation of a markedly different copy number of R45-FIKK kinase genes in *P. falciparum *as compared to *P.y. yoelii *or *P. vivax *is in line with distinct sets of subtelomeric multigene families in different *Plasmodium *species. This is further substantiated by the observation that the *P. falciparum *R45-FIKK kinase genes are situated at or close to subtelomeric breakpoints of synteny between *P. falciparum *and *P. yoelii *[2]. The association of most R45-FIKK kinase telomeric paralogs with a DNA J / *resa *paralog and with additional paralogs from families coding for exported hypothetical membrane proteins or proteins involved in lipid metabolism suggests a structured complex interacting with and signalling from the host cell membrane. It is possible that different subtelomeric gene families execute this function in species such as such as *P. vivax, P. knowlesi *or *P.y. yoelli *that do not possess the specific adhesion properties associated with expression of erythrocyte variant antigens observed in *P. falciparum*.

## Conclusion

The new R45-FIKK kinase gene family codes for hybrid proteins with a variable N-terminal domain associated with a conserved, novel protein kinase domain. The presence of most key amino acids for phosphotransfer and the remarkable sequence conservation suggest an enzymatic function. The restricted occurrence of R45-like genes in Apicomplexan parasites and the distance from known kinase families makes a case for consideration of this family as a potential drug or vaccine target. The family shows considerable expansion in *P. falciparum*. All paralogs code for proteins with canonical export signals and most are subtelomeric, clustered with a subset of multigene families coding for exported proteins. This suggests that expansion of this family might contribute to *P. falciparum *virulence.

## Methods

### Retrieval of homologous sequences in *P. falciparum*

The deduced protein sequence of PFD1175w was used for a tblastn search against the *P. falciparum *annotated proteins in the PlasmoDB database version 4, and verified for version 4.3 for which genome coverage is 18× . (Note that PFD1175w has been annotated as R45 trophozoite-like antigen in the genome sequence, whereas PF10_0160w, a paralog without repeats was annotated as trophozoite antigen R45 [1]. This is inappropriate, since the only gene that encodes the hexapeptidic repeats originally described for the gene named R45 by Bonnefoy et al [4] is PFD1175w. The PFD1175w repeat region alone did not yield any significant hit apart from PFD1175w either by blasting the whole repeat region or by scanning a single repeat motif against the database. Blasting the PFD1175w protein sequence without repeats, however, resulted in identifying 20 additional annotated proteins with high homology from amino acid 248 up to the end of the ORF, with E-values ranging from 6.8e-109 for PF10_0160 to 2.6e-28 for PFC0060c, with the next best hit down to 7.3e-05.

The primary structure of the R45-like proteins was verified by comparing and aligning the prediction alternatives made by GlimmerM, Genefinder and FullPhat (PlasmoDB). Sequences were used as annotated in PlasmoDB for the following 8 proteins: PFD1165w, PFD1175w, PFE0045c, PFI0100c, PFI0110c, PFI0120c, PFI0125c, PF10_0380. For PFI0105c, PFI0115c and MAL13P1.109, preference was given to the alternative sequence prediction chr.9 glm31, chr9.glm33 and chr13phat_225, respectively, that includes the 3^rd ^exon that is highly conserved in all other R45-like protein paralogs. For PF10_0160, the alternative sequence prediction chr10gen334 was preferred, because the boundary between exon 2 and 3 resulted in a perfect alignment with other family members, whereas the annotated version aligned with gaps. For PFA0130c, PFC0060c, Mal7P1.175, PFI0095c, PF11_0510 and PFL0040c, preference was given to the chr1.glm_35, chr3.glm_19, chr7.glm_316 and chr9.glm_29, chr11.glm_542 and chr12.glm_14 predictions respectively, because they include a signal sequence to the putative N-terminal domain. For Mal7P1.144, a 5' exon was predicted manually resulting in a three exon protein with an N-terminal signal sequence. Mal7P1.175 (chr7.glm_316) was manually curated by fusing the first two exons and considering the intron sequence as coding. This allowed a perfect alignment with its paralogs, although including one internal stop codon. Likewise, the two tandem chromosome 14 genes, annotated PF14_0733 and PF14_0734, were fused together, referred to as PF14_0733+4, resulting in one full R45-like protein interrupted by one stop codon. The identical asexual stage mRNA expression pattern of both genes (DeRisi Lab Malaria Transcriptome Database ) strongly suggests that they indeed form a single transcription unit. The gene names for all paralogs but PF14_0733+4 were kept as annotated by PlasmoDB, even when an alternative prediction was preferred, resulting in a total of 20 R45-like proteins in the 3D7 *P. falciparum *genome. Signal sequences were predicted using the signalP 3.0 server [10].

Note that MAL13P1.109 and the flanking two genes were no longer retrieved from the PlasmoDB version 4.3 released November 2004. Since the reason for this is unclear, we decided to keep including this gene within the family described here.

### Retrieval of R45-like genes from other *Plasmodium *species, *T. gondii *and *C. parvum*

R45-like protein orthologs from other species were identified using a tblastn search of the protein kinase domain of PFD1175w against the genomic sequences of all *Plasmodium *species in PlasmoDB. This identified six highly matching contigs (E- value <10 exp 40) as follows: Pg_c000319933.Contig1 from *P. gallinacaeum*, Pk_2154b11q1c from *P. knowlesi*, Pv_4038 from *P. vivax*, chrPyl_00951 from *P.y. yoelii*, Pb_5155 from *P. berghei *and Pr_4e04q1k from *P. reichenowi*. The same tblastn search was also repeated in the genomic databases from the Wellcome Trust Sanger Institute  for *P. berghei*, *P. knowlesi, P. gallinacaeum and P. reichenowi *sequences and the Institute for Genomic Research  for *P. vivax *and *P. yoelii *sequences. Contigs with the identical sequence information as those obtained from PlasmoDB were obtained for five species, however six highly matching hits were retrieved in the *P. reichenowi *genomic database. No hits were obtained by blasting against other protozoan parasites in the above mentioned resources. The *T. gondii *contig TGG994720 was retrieved from the Toxoplasma Database [ToxoDB, release 3.0, ] and the *C. parvum *contig AAEE01000010.1 was retrieved from the National Center for Biotechnology Information . BlastP analysis of PFD1175w against the NRprot database did not give a hit in any additional species.

R45-like orthologs were predicted as follows: the *C. parvum *ortholog annotated as EAK87586.1 [3] was named CpR45. The gene model proposed for the *P.y. yoelii *ortholog [2] was modified. The two genes annotated as Py03325 and Py03326 which are separated because of a one nucleotide frameshift were fused to one gene and named PyR45. The resulting two exon structure with most of the sequence encoded by exon 1 and the conserved exon 2 (corresponding to exon 3 in the *P. falciparum *orthologs) is supported by the EST data. Two exon orthologs in *P. vivax*, *P. knowlesi*, *P. berghei, P. gallinacaeum *(PvR45, PkR45, PbR45 and PgR45) were predicted accordingly.

Six R45-like proteins were identified in *P. reichenowi *(PrR45-1 to 6) from contigs 3502696.c000125078, c000024654, c000125213, c000126950, c000124557, c000128208, respectively. Their very close homology to specific orthologs in one or the other species was exploited to predict their exon-intron boundaries. The *T. gondii *R45-like protein, TgR45 was predicted using the overlapping ESTs TgEST_95056840, TgEST_100112765, TgEST_95056367. The N-terminal exon(s) are not yet identified.

The databases were last checked for the presence of new hits by December 9, 2004. By then, the genome of *P.y. yoelii *was as published in 2002, namely with 5× coverage and short contigs [2], the genome of *C. parvum *was published with 13× coverage and 1–3 large contigs per chromosome [3]. Genome sequence of the other species was in progress, with mostly short contigs and the following coverage rates: *T. gondii *(10×), *P. vivax *(10×), *P. knowlesi *(5×)*, P. berghei *(3×), *P. gallinacaeum *(3×)* and P. reichenowi *(1.5×).

### Analysis of chromosomal organisation in *P. falciparum *and possible synteny with other species

Analysis of the genes localised within 40 kb on either side of the various R45-like paralogs was done using the published chromosomal maps [1] and the PlasmoDB version 4.3 annotation. Ortholog groups were systematically looked for each gene, and sequence alignments inspected. For each family, only paralogs with high homology along >60% of the sequence were considered. In case a *P.y. yoelli *ortholog was present, the neighbouring genes were analysed for possible synteny groups with *P. falciparum*. The same was done for the *P. vivax *genes located within contig Pv_4038.

### Sequence alignment and phylogenetic tree

The conserved regions of all R45-like proteins were aligned by ClustalW [11] using the matrix PAM30 and adjusted manually where necessary. Four well characterised catalytic domains of protein kinases, namely protein kinase A (PkA), Jun-kinase 1 (Junk1), fibroblast growth factor receptor 1 (Figure 1) and extracellular regulated kinase 1 (ERK1) were used as a scaffold for the alignment of R45-like proteins to the 12 subdomains as provided in [12]. The *P. falciparum *protein kinase 5 (PfPk5), the only *Plasmodium *protein kinase which has been thus far structurally characterised, was included as an additional reference protein kinase [13]. For the phylogenetic tree, the kinase domains of 32 R45-FIKK proteins were taken and gaps between the subdomains were removed. Phylogenetic relationships were inferred by the neighbour joining method [14] with the *T. gondii *protein TgR45 as outgroup. The reliability of the trees was assessed by the bootstrap method [15].

## Authors' contributions

AS performed the database analysis for R45 paralogs and orthologs in the various species, reannotated the genes, performed the alignments and constructed the phylogenetic tree. OP searched for PEXEL export motifs, performed database mining for chromosomal localisation, analysed the conservation of the multigene families located in the vicinity of R45-FIKK like *P. falciparum *paralogs, generated the chromosomal map and investigated possible synteny in other species. OP and AS carried out data mining for expression profiles throughout the life cycle. AS and OP drafted the text of the manuscript. OP finalised the revised version.

## Supplementary Material

Additional File 1 Multiple sequence alignment of the kinase domain from all R45-FIKK kinase proteins identified.
Click here for file

Additional File 2 Multiple sequence alignment of the N-terminal region of R45-like proteins in *P. falciparum *and its orthologs in *P. reichenowi*. Click here for file

Additional File 3 Multiple sequence alignment of the N-terminal region of R45-FIKK kinase orthologs from six *Plasmodium *species. Click here for file
