# Supplementary material for: A new Apicomplexa-specific protein kinase family : multiple members in Plasmodium falciparum, all with an export signature
Source: BMC Genomics. 2005 Mar 7;6:30. doi: 10.1186/1471-2164-6-30 (PMC1079819; doi:10.1186/1471-2164-6-30)
Supplement: Additional File 1 — Multiple sequence alignment of the kinase domain from all R45-FIKK kinase proteins identified. [file 1471-2164-6-30-S1.rtf]

Additional file 1
Multiple sequence alignment of the kinase domain from all R45-FIKK kinase proteins identified. 
The single letter amino acid code is used; gaps are indicated by dashes, number of excluded residues is indicated in brackets. The kinase domain of the human protein kinase A (PkA, accession AAH39846), human fibroblast growth factor receptor 1 (Flg, accession NP_075594), rat extracellular signal regulated kinase (ERK1, accession NP_059043), human Jun kinase 1 (Junk1, accession P45983) and the P. falciparum protein kinase 5 (Pfpk5, accession NP_705452) was used as a scaffold for the alignment of R45-FIKK kinase proteins in eleven distinct protein kinase subdomains (Roman numerals). In these canonical kinase domains, the twelve amino acids that are nearly invariant throughout the kinase superfamily are highlighted in colour. Within the R45-FIKK kinase proteins, a 50 % identity threshold was set for colour highlighting the amino acids in a conserved position.


Subdomain                                               I                                                   II        
                                              43                   64                                    65           
                                              |                     |                                    |            
cAPKa        ---------------------------------FERIKTLGT-GSFGRVMLVKHKE------------------------------------TGNHYAMK 30  
Jnk1         ---------------------------------YQNLKPIGS-GAQGIVCAAYDAI------------------------------------LERNVAIK 30  
Flg          ---------------------------------LVLGKPLGE-GCFGQVVLAEAIGLDKDKPN-----------------------------RVTKVAVK 37  
ERK1         ---------------------------------YTQLQYIGE-GAYGMVSSAYDHV------------------------------------RKTRVAIK 30  
PfPk5        ---------------------------------YHGLEKIGE-GTYGVVYKAQNNYGET-------------------------------------FALK 29  

                      10        20        30        40        50        60        70        80        90       100    
             ....|....|....|....|....|....|....|....|....|....|....|....|....|....|....|....|....|....|....|....|
MAL7P1.144   IYNWKIGKECFMKKLDSVH-NFEMNGVNYYDFNLISIPTIGY-SKSSKRLQLMYKTDVIYGENE----------------------NDKNNLKKKKLFLK 76  
PrR45-4      IYNWKIGKECFMKKLDGVH-NFEMNGVNYYDFNLISIPTIGY-SKSSKRVQLMYKTDVIYGENL----------------------NDENNLKKNKLFLK 76  
PF10_0160    IYNWNLGKKSLCNMLENSD-NYYINGVKYSDWKLTSIPTHGY-NKEGGRVQEMFKTFVSS--------------------------KEGDGSDRVGLFIK 72  
PgR45        IFNWKLAQKALIKMLHSAH-NFYFNGVKYSDWDLTSIPTLGF-SKSSNRVQQMFKATVPS--------------------------KDGNSKNEVKLFIK 72  
PkR45        IYNWKLAQKSLLKMLHSAH-NYYFNGVQYADWQLTSIPTLGF-SKSSNRVQQMYKAVIPS--------------------------KDGSGKNEVKLFLK 72  
PvR45        IFNWKLAQKSLLKMLHSAH-NYYFNGVEYADWQLTCIPTLGF-SKSSNRVQQMYKAVIPS--------------------------KDANSKNEVKLFLK 72  
PrR45-2      IFNWKIAKKSLMKMLHSAH-NFYFNGVKYSDWKFTCIPTLGF-SKSSNRVQQMYRAFVVP--------------------------KDSNVKKEVKLFIK 72  
PbR45        IFNWKIAQMSLLKLLHSAH-NFHFNNVKYHDWKFICIPTLGF-SKSSNRVQQMYKAIIPS--------------------------KDGNSKNDVKLFIK 72  
PyR45        IFNWKIAQMSLLKLLHSAH-NFHFNNVKYSDWKFICIPTLGF-SKSSNRVQQMYKAIIPS--------------------------KDGNSKNDVKLFIK 72  
MAL13P1.109  VYNWELGQKSLIKMLDYAD-NFYFNGVKYSDWKLTSMRRFNL-NNNVLKDHKTYKSIINS--------------------------KKGNDMKKVKLFIK 72  
PFC0060c     IFNWHLGKISVLKMLDNAE-NFSIGGVKYKDWDLIPILPPNN-NKNDKKVHKMFKASISA--------------------------RYGLKNKTMKFFIK 72  
PrR45-5      ------GKISVLKMLDNAE-NFSIGGVKYKDWDLIPILPPNN-NKNDKKVHKMFKASISA--------------------------RYGLKNRTMKFFIK 66  
PFI0105c     IFNWDIGKETLLKMFDMSE-NFEINDVNYESWILHKVPTKNY-SERSGRVQEMFKVVINS--------------------------KDGSG-NDIRLFVK 71  
PF14_0733+4  VYNWTLGKKALSKMLHYDDDNFSINGVKYPDWKLKPIPTIGY-SKKSGRVQEMYTTVIKG--------------------------NPDENTEDVKLFIK 73  
PFE0045c     IYTWALGKDALSDKLYSSS-NYSLNGIQYNYWRLSAIPTINY-SVRTSRVQKMFKTIIDP--------------------------KKENAKDQVKAFIK 72  
PFD1165w     LYNWKLGKSILGKMINSTE-DFSINGISYKDWTLNTIPDIGY-SQNNRRSQEVFKTEVKS---------------------------KDENTESVKLFIK 71  
PF10_0380    VYNWESGKKALGKLLSNSD-KLSVNNIKYSEWNLERIPTLGF-CKEDDRVQEMFKASIMG--------------------------NNDDSTKEVKFFIK 72  
PrR45-1      VYNWKSGKKALGKLLSNSD-NLSVNNIKYSEWNLERIPTLGF-CKEDDRVQEMFKASIMG--------------------------NNDDSTREVKFFIK 72  
PFD1175w     VYSWELGKEALTKLLGCSN-KLSVNNVKYSDWKFKSIPTVGF-SKDGDRLQEMYKASVCS--------------------------YTEECQGEVYLFVK 72  
PFA0130c     IYTWDLGRESLGKLLDSSK-NFSINGVKYDDWETTPIPTCGA-SRVMEKCQKMYKVVIKPKEKDDKVDNKIKMGNCNDCDKIKGKDNNEEEGDEIKLFMK 98  
PFL0040c     IYNWNLGKESLVKFLGFSD-YFKINGVKYSDFELTSIPIIGE-NKSKGRVQEMFKTVIPS--------------------------NDGDPAKEVKLFIK 72  
PF11_0510    IYNWIEGYKSLVKMFGLSN-NFSINGVKYSDWKLIPISFIEY-NKKKFRVQEMFKTVITS--------------------------KNDDNKNNISLFIK 72  
PFI0095c     IYNWKLGKDSLLKMLCMSK-DYSINGVNYENWKLSPIDFGDMDDISKKKKKNMYKSIICS--------------------------PKGNSINNTKVFIK 73  
MAL7P1.175   LYNWELGKQCLLKMLDFSY-NFCVYGMNYDVWELKRITTNNC-EIGSSRVHKMYETFISS--------------------------KNG---NGIRLFIK 69  
PFI0110c     IYNWELGNKTVNEFLGHAD-NFEMNGVNYYDWKLTTIPTKGY-NTERGRGHEMFKAVIPS--------------------------NGGDKKDDITLFIK 72  
PrR45-3      IYNWELGNKTVNEFLGHAD-NFEMNGVNYYDWKLTNIPTKGY-NTERGRGHEMFKAVIPS--------------------------NGGDKKDDITLFIK 72  
PFI0115c     IYNWKLGIRCLNKYLDLSK-RFEIKGVNYDEWNLTYLPLNSF-CNKKERIHKIFETRISY--------------------------NNVNKKSELKLFIK 72  
PFI0120c     IYNWELGKACLSKYLGHSN-NYFLNGVKFDEWKLVSFRDRYY-NERKGRVHKILKTKITG--------------------------NDGNNKRELKLFIK 72  
PrR45-6      VFNWELGKESLKKRLGCIN-NFQINGVYYKNWILKNITFGNS-SNPYHSSKNVYKGIIPI--------------------------NNNYNVDEVKVFIK 72  
PFI0125c     VFNWELGKESLKKRLGCTN-NFQINGVYYENWILKNINFRNS-SNSHYSPKNVYKGIIPM--------------------------NNNYNVDEVYVFIK 72  
PFI0100c     LFNWELCKYHISNRLGKAK-EYSIGGVNYEKWDLYSIKNENY-NESGGRNHEMFSTVISS--------------------------KSGFRKKKVKLFIK 72  
TgR45        FPVWVLCRELIARRTYRAK-TYVLDGVPFDRWRLQVVPTMGA-STTSTRCQRMWKGRLPS---------------------------------GRAVFVK 65  
CpR45        CYTWKEAKQLISKLKSSTK-LYKFNGVPFSDWAHEKIPTLGA-SPTSCRVQEMFKSRIYL---------------------------EKNNEVYATLFIK 71  


Subdomain        II                       III
                      83            84           98
                       |            |             |
cAPKa        ILDKQKVVKLK------------QIEHTLNEKRILQAV-------------------------------------------------------------- 56  
Jnk1         KLSRPFQNQT-------------HAKRAYRELVLMKCV-------------------------------------------------------------- 55  
Flg          MLKSDATEK--------------DLSDLISEMEMMKMIG------------------------------------------------------------- 62  
ERK1         KISPFEHQT--------------YCQRTLREIQILLGF-------------------------------------------------------------- 54  
PfPk5        KIRLEKEDEG-------------IPSTTIREISILKELK------------------------------------------------------------- 55  

                     110       120       130       140       150       160       170       180       190       200    
             ....|....|....|....|....|....|....|....|....|....|....|....|....|....|....|....|....|....|....|....|
MAL7P1.144   KVPANLWIEQYKLMKEYDGEYVYSGENYVMEFLVLSFLDTYHPN-ICPKLYKILYEPPNK--------------------------EYIKDENKKFQNID 149 
PrR45-4      KVPANLWIEQYKLMKEYDGEYVYSGENYVMEFLVLSFLDTYHPN-ICPKLYKILYEPPNK--------------------------EYIKDENRKFQNID 149 
PF10_0160    KIPVDIWVKQFELMNAYNGEYVLCGENYVMEATTLAFLNEYYPG-ITPKLYKILYEPEKK---------------------------------------- 131 
PgR45        KIPIYIWVKQFNLMNEYDGEYVTDGENFVMEAAALAFLNEYHQG-ITPKLFKVLYEPESK---------------------------------------- 131 
PkR45        KIPIYIWIKQYNLMNEYDGEYVTDGENFVMEATSLAFLNEYHPG-ITPKLHRILYEPDGK---------------------------------------- 131 
PvR45        KVPIYIWIKQYNLMSEYDGEYVTDGENFVMEATSLAFLNEYHPG-ITPKLHRILYEPDGK---------------------------------------- 131 
PrR45-2      KIPIYIWVKQFNLMNEYDGEYVTDGENFVMEATALAFLNEYHPG-ITPKLYKILYEPDNK---------------------------------------- 131 
PbR45        KIPIYIWVKQFNLMTEFDGEYVTDGENFVMEAASLAFLSEYHPR-IAPKLHKILYEVDNT---------------------------------------- 131 
PyR45        KIPIYIWVKQFNLMSEFDGEYVTDGENFVMEAASLAFLSEYHPR-IAPKLHKILYEVDSN---------------------------------------- 131 
MAL13P1.109  KIPIDIWVEQFNLMKKYEGEYLIDKENYVMEAVSLAFLNEYYPG-ITPKFYKILYESDKN---------------------------------------- 131 
PFC0060c     KIPMDTWVKQYNLMTEYDGEYLLAGENAVMEAMALAFLNEYHPN-IAPKFYKLLYEEDNK---------------------------------------- 131 
PrR45-5      KIPVDIWVKQYNLMTEYDGEYLLAGENAVMEAMVLAFLNEYHPN-VAPKFYKLLYEEDNK---------------------------------------- 125 
PFI0105c     KIPIKVWVKQYNLMKKYNGEYVFGSENFIMEAMALSFLTEYYPG-IAPKLYKVLIEPDNI---------------------------------------- 130 
PF14_0733+4  KVPIEIWVKQFDKMARYRGEYLVNAENFVMEAVASAFLTEYHPG-ITPKLYKILYDP------------------------------------------- 129 
PFE0045c     KIPVDIWVKQFHSMNEYDGEFLVGGENFVMEAVTSAFLTKYHPG-ITAKLYALLYEPYRK---------------------------------------- 131 
PFD1165w     KVPASIWVRQYIILNEHKGEYSLGEENFVMEAISLAFLNKYYPG-IAPKFYGILYESPDK---------------------------------------- 130 
PF10_0380    KIPIDIWLKQYKLMNEYDGEYLLDGENFVMEAVASAYLSEHYPG-LIPKLYKVVYEPVNN----------------NTNNNNDEKNNKKHSNDKKSKEDN 155 
PrR45-1      KIPIDIWLKQYKLMNEYDGEYLLDGENFVMEAVASAFLSEHYPG-LIPKLYKVVYEPVNN--------------NNNNNNKNDEKNNKKYSNDKKYKEDN 157 
PFD1175w     KIPIEVWIRQYNLMNDNDGEYLLDGENFIMEAVACAYLSEHYPG-LTPKLYKVLYEPECANCNEEDKNMSENN------(HKSDHN)X90------KNNN 159 
PFA0130c     KVPIDVWVKQYDLMKEYDGEYLSVGENFVMESVVLAFLNEYHPG-IAPKFYKFLYEPDMN---------------------------------------- 157 
PFL0040c     RIPVEWWIKQFNLMEKYDGEYLVKAENYVMEGVALSFLSEHHPG-IAPKLLKILYDGKNV---------------------------------------- 131 
PF11_0510    KIPVDIWLKQFEMMELYNGEYLVNAENYVMEASILAFLNEYYQGFIAPKLYKILYEENYE---------------------------------------- 132 
PFI0095c     KINIKDWLKLFNCMEKYDGEYMYTKDNFVMEAVALSFLEEYHKG-ITPKLHKILFEPDDN---------------------------------------- 132 
MAL7P1.175   KIPISAWVKQYKLMNEYEGEYIINAENYVMEAVALSFLNEYYPG-IAPKLYRVLFQPDVH---------------------------------------- 128 
PFI0110c     KIPINLWIKQYDLMNMLYGEYLMGGENFVMEVMVYAFLTKYYPG-ISPKLYKVLFVPEDR---------------------------------------- 131 
PrR45-3      KIPINLWIKQYDLMNMLYGEYLMGGENFVMEVMVYAFLTKYYPG-ISPKLYKVLVVPEDR---------------------------------------- 131 
PFI0115c     KIPIDIWLKQFEMMQMYNGEFIENGENFVMEAIVLSFLNEYYPN-ICPKFYRLLYEPHYY---------------------------------------- 131 
PFI0120c     RIPIDMWLKQFEMMDTYNGEYIICAENFVMEAIVLSFLSAYHPG-IGPKFYKLLFNPDNY---------------------------------------- 131 
PrR45-6      KIPIDKWLTQYEKMELYNGEYVLNGENYVMEAVVSAFLSDYHPG-ISPYFYKLLYEPVDS---------------------------------------- 131 
PFI0125c     KIPINKWLNQYEKMELYNGEYILKGENYVMEAVVSAFLSEYHPG-ISPHFYTLLYEPLDYNENGNENGNENGNGDENGNENGNENGNENGNENGNENGNE 171 
PFI0100c     KVPLNSWIELYNKMDIYHGEFLDGAENFVMEAMVSLFLNKYHPG-ITPKFYNLLYESEND---------------------------------------- 131 
TgR45        KVPSSVWEQQWRLTQRYKGFFLTDGENFVGEAAISAFLTDCGPACAAP-LLAVLHE-------------------------------------------- 120 
CpR45        KIPRNIWSKQWEMHEIWDGDYVTDGEDFVMEAAALAFLQNHSVG-IAPRLYAILEHCEDN---------------------------------------- 130 


Subdomain                                               IV                                   V
                                                  99          113                 114                  137
                                                  |             |                 |                      |
cAPKa        -------------------------------------NFPFLVKLEFSFKDN-----------------SNLYMVMEYVPGGEMFSHLRRIGR------- 95  
Jnk1         -------------------------------------NHKNIIGLLNVFTPQKSLEEF-----------QDVYIVMELMDA-NLCQVIQME--------- 97  
Flg          -------------------------------------KHKNIINLLGACTQD-----------------GPLYVIVEYASKGNLREYLQARRPPGLEYCY 108 
ERK1         -------------------------------------RHENVIGIRDILRAPTLEAM------------RDVYIVQDLMET-DLYKLLKSQQ-------- 96  
PfPk5        --------------------------------------HSNIVKLYDVIHTK-----------------KRLVLVFEHLDQ-DLKKLLDVCEGG------ 93  

                     210       220       230       240       250       260       270       280       290       300    
             ....|....|....|....|....|....|....|....|....|....|....|....|....|....|....|....|....|....|....|....|
MAL7P1.144   DFVKYMEDIIEHNKRNNANNNVDNNNNI---------HNHKNNIN-YCITNSDNKHDNNNNDNNSDNNCGYVVMVSEYYGE-DIFDFIIKRRKNIFLK-- 236 
PrR45-4      DFVKYMEDIIEHNKRNSANNNVDNNN-I---------HNHKNNIN-YCITNSDNKHDNNSNDNSSDNNCGYVVMVSEYYGE-DIFDFIIKRRKNVFLK-- 235 
PF10_0160    ----------------EYDIEQNTPDCM---------FHDLNVFN-DILS---ARLKCNMN--------GYIVIISELFGE-DIYTYLTKQKKKNIFA-- 191 
PgR45        -----------------HFNEYMAPKAM---------FNNLNLFN-NILT---ERLKCNIN--------GNIVIVSEFFSE-DILDYIDRRQKKYNMK-- 190 
PkR45        ----------------EPSEYNIPPKSM---------FNSLALFN-DILT---ERLKCNIS--------GNIVIVSEFFSE-DILDFIDRRQKKLNMK-- 191 
PvR45        ----------------QPNECNIPPKSM---------FNSLTLFN-DILT---ERLKCNIS--------GNIVIVSEFFSE-DILDFIDRRQKKLNMK-- 191 
PrR45-2      -----------------YINEGLSQKSM---------YNNLNVFN-DMLS---ERLKCNAG--------GNIVIVSEFFNE-DILDFIDRRQKKYNMK-- 190 
PbR45        --------------NLDGSNNNIPPESM---------FSNLTVFN-NVLE---ERLKNNIN--------GNIVLVSEFFSE-DILDFIDRRQKNFNMK-- 193 
PyR45        --------------NLDDSNNNILPESM---------FSNLTIFN-NVLG---ERLKNNIN--------GNIVLVSEFFSE-DILDFIDRRQKNFNMK-- 193 
MAL13P1.109  -----------------NMNEKNCKKYK---------FQDLNELN-DILT---KKLENNIN--------GNIVLISEFFGE-NVFDYIKRKKNTLFVVSD 192 
PFC0060c     -----------------NSSDCILDNEI---------FYDLNLFN-DFLC---EKLNTNIN--------GNIVMISEYYGE-DIFDFILRETEGFNTF-- 190 
PrR45-5      -----------------NCTDCILDNEN---------FYDLNLFN-DFLC---EKLNTNIN--------GNIVMISEYYGE-DIFDFILRETEGFNTF-- 184 
PFI0105c     -----------------YYYKGITKEKM---------FDNMNTLN-EILS---KGIKDDIN--------GNIVIVSELFGK-DIKKFLFTENENILAI-- 189 
PF14_0733+4  ----------------IXENKKSLHKIA---------FNDLGAFN-YILR---NRLKSNIE--------GNIVIISELYGQ-DIFNYIDKKRLDIGMDDD 191 
PFE0045c     -----------------YIQEDSPPKSS---------FENIDSFN-EMLE---EKIKNNKK--------GNVVLIYELFGE-SLFTNLVKSRRKPILK-- 190 
PFD1165w     ---------------PNDNVCFSFGRGK---------CNNLKKFN-DMLI---NQLQLNNK--------ANVIMISELYGE-DVFKYVKNKRKEGCFG-- 191 
PF10_0380    DDMGYHTGDENDIFEDCNDSNSDRPIDG---------YDHLKKFN-DMLT---KQLNNNKK--------GYVVFISELYGQ-DLFQYINNKNENKETV-- 231 
PrR45-1      DDMGYHTGDENDIFEDCNDSDSDSSIDD---------YDPLNKFN-DMLG---KQLDNNKK--------GYVVFVSELYGQ-DLFQYINNKNENKETV-- 233 
PFD1175w     NNKDNKNDDNDDSDASDAVHEDIELLES---------YSDLNKFN-EMLT---EQLNKNKD--------GYVVLVTELFGE-DLFQYINKRNENEDTR-- 235 
PFA0130c     -----------NDLNNKCDEKNKYVLHI---------DRNLDTFN-ERLR---EEVNNNKK--------GYVVMVCEFFGE-DIFDYTISEKERMGTE-- 222 
PFL0040c     -------------------NHDIMEEYK---------FKDIYEFN-NMLI---ERINNNMD--------GYIVMVSELFGE-DLFDFNKRFTKEKSDVR- 189 
PF11_0510    --------ENNKENMFPPYMFNEKKELN---------INNLHEFK-NFLK---ERINKNVN--------GYIVIVSELYGQ-NVFEYIEKRQKENNNI-- 200 
PFI0095c     -----------------EFYENVSSDYM---------FSSLDNFN-KILS---NGLEKNLD--------GNIVIVSELYGD-DIKNYKKKMRKVYPKV-- 191 
MAL7P1.175   -----------------YIGGEFPQENI---------FQDLDTFN-SVLT---NELESNMN--------GYIIIVSEYFGE-NINEYIKRQRKKMFSI-- 187 
PFI0110c     -----------------CNFLNISTSHM---------SKDIISFN-YILN---RMLIKNMK--------GYVVMVSEYYGK-DTYKYLRKKGGIYRD--- 189 
PrR45-3      -----------------SNALNISTSYM---------SKDIISFN-YIIN---RMLIKNMK--------GYVVMVSEYYGK-DTYKYLRKKGGIYRD--- 189 
PFI0115c     -----------------FNLKSISCFEN---------INDINIFN-NLLR---EHLKMNMI--------GYVVMISEFFGE-DLENYIYNIRNKKTYK-- 190 
PFI0120c     -----------------YIDENNSSMNN---------INDISLFN-KLLS---DRLRMNMS--------GYILMVSESFGE-DLETYLCTVNNKKIFK-- 190 
PrR45-6      ---------------NESENENRSDENI---------IPNLNVFN-DILK---EQNKLKRI--------SNIVMVFEFFGE-DLDCYIKKMCIKGYST-- 192 
PFI0125c     NGNGNGVENGNENGVENGNENGNAIRSY---------ENNIPNLN-VFNDILKEQNKHKSI--------GNIVMVFEFFGE-DLDCFMNRMCIKGYST-- 250 
PFI0100c     -----------------YSELKGLNELM---------FCDIDIFK-NELI---KIRNRNKK--------GYVVMIWEFFGQ-NLKEFLHSEKENL----- 187 
TgR45        -------------------------DGN---------YPEGPSVT-RTDS---EATETGST-------TSRVVLVNQVFGQGDLLDFFDSADPEV----- 170 
CpR45        -------------------CNDINSNKE--------SKIQYRNIA-NRNA---CDYLLSKD-------TTHIILVSEHYGE-DLLDYLDKCEKKNRN--- 188 


Subdomain                          VIa                                 VIb
                         138                  160               161            178
                         |                      |               |                |
cAPKa        ------------FSEPHA-RFYAAQIVLTFEYLHSL---------------DLIYRDLKPENLLIDQQG------------------------------- 136 
Jnk1         ------------LDHERM-SYLLYQMLCGIKHLHSA---------------GIIHRDLKPSNIVVKSDC------------------------------- 138 
Flg          NPSHNPEEQ---LSSKDL-VSCAYQVARGMEYLASK---------------KCIHRDLAARNVLVTEDN------------------------------- 158 
ERK1         ------------LSNDHI-CYFLYQILRGLKYIHSA---------------NVLHRDLKPSNLLINTTC------------------------------- 137 
PfPk5        ------------LESVTA-KSFLLQLLNGIAYCHDR---------------RVLHRDLKPQNLLINREG------------------------------- 134 

                     310       320       330       340       350       360       370       380       390       400    
             ....|....|....|....|....|....|....|....|....|....|....|....|....|....|....|....|....|....|....|....|
MAL7P1.144   ------------IRRKDK-INILHACLKLLARLHDA---------------GLCHLDLTPDNILISKSM------------------------------- 277 
PrR45-4      ------------IRRKDK-INILLACLKLLARLHDA---------------GLSHLDLTPDNILISKSM------------------------------- 276 
PF10_0160    ------------LHSYMKRKKILFECLNVLRKLHDA---------------GLCHLDISPQNILMSYNF------------------------------- 233 
PgR45        ------------ISNNEK-SYILYQCLKLLIRLHDA---------------GLSHLDLTPENILISDNY------------------------------- 231 
PkR45        ------------ISNNEK-SYILYQCLKLLIRLHDA---------------GLSHLDLTPENILISESY------------------------------- 232 
PvR45        ------------INNNEK-SYILYQCLKLLIRLHDA---------------GLSHLDLTPENILISDNY------------------------------- 232 
PrR45-2      ------------ISNNEK-SFILYQCLKLLIRLHDA---------------GLSHLDLTPENILISDNY------------------------------- 231 
PbR45        ------------ISNNEK-SYILYQCLKLLIRLHDA---------------GLSHLDLTPENILISDNY------------------------------- 234 
PyR45        ------------ISNNEK-SYILYQCLKLLIRLHDA---------------GLSHLDLTPENILISDNY------------------------------- 234 
MAL13P1.109  ------------ISNEDK-KKILYNSLNLLMRLHNA---------------GLTHLDLSPDNMLISPKNY------------------------------ 234 
PFC0060c     ------------LNKNKK-KKILYESLHLLVKLHDA---------------GFSHLDLSPENILISDKY------------------------------- 231 
PrR45-5      ------------LNKNKK-KRILYESLHLLVKLHDA---------------GFSHLDLSPENILISDKY------------------------------- 225 
PFI0105c     ------------IDNNNK-KKYLIESLKLLVRLHEA---------------GLAHLDFTPENILIKKNG------------------------------- 230 
PF14_0733+4  DDDLV-------LTVEEK-KSILYKALNLYTRLHEA---------------GLAHLDLSAENVLIDENN------------------------------- 237 
PFE0045c     ------------NRYAKK-KKIIYDSLNLLIRLHDA---------------GLTHLDFTPENILISENN------------------------------- 231 
PFD1165w     ------------NNLEEK-KKILHESLKLITTLHET---------------GLSHLDISPENILIGNNC------------------------------- 232 
PF10_0380    ------------VSVEEK-KKIMKECLKLLIKLHNA---------------GLAHLDISPENILISNNS------------------------------- 272 
PrR45-1      ------------VSVEEK-KKIMKECLKLLIKLHNA---------------GLAHLDISPENVLISDDS------------------------------- 274 
PFD1175w     ------------VRDEDK-KIIMFEFLKLLIKLHNA---------------GLVHLDISPENILIENNG------------------------------- 276 
PFA0130c     ------------EWFEDV-KKILFKSLKLLIRLHDV---------------GITHLDLTPENVLITKNF------------------------------- 263 
PFL0040c     ------------NSDEFK-KELLYKCLRLLVRLHSA---------------GLSHLDLTAENVLITDDY------------------------------- 230 
PF11_0510    ------------LSDREK-KKILYECLKLLIKLHNV---------------GIAHLDISLENILMTENY------------------------------- 241 
PFI0095c     ------------FNKKSK-KKILFECLKLIDKLHDT---------------GLSHLDFTPENILISDNF------------------------------- 232 
MAL7P1.175   ------------GRKKKK-KKLLYNCLNLLRKLHNA---------------GLSHLDFTSHNILISDKH------------------------------- 228 
PFI0110c     ------------ISENER-KKIIHEWIKLVSRLHDT---------------GLSHLDISPENTLIGENH------------------------------- 230 
PrR45-3      ------------ISENER-KKIIHEWIKLVSRLHDT---------------GLSHLDISPENTLIGKNH------------------------------- 230 
PFI0115c     ------------FKKNDK-KKIMLECLKLINKLHQV---------------GICHLDFSIDNILISKNG------------------------------- 231 
PFI0120c     ------------SRKKNK-KKLLFESLKLINKLHQA---------------GICHLDFTLDNILISKNG------------------------------- 231 
PrR45-6      ------------LGRKGK-KKIMLSSLKLINRLHKI---------------GLCHLDISLENILMKDNY------------------------------- 233 
PFI0125c     ------------LGRKAK-KKIMLSCLKLINRLHKI---------------GLCHLDISLENILMQDNY------------------------------- 291 
PFI0100c     ------------VITKER-KKILFECLKLINKLHKA---------------GLTHLDISPENILIGENY------------------------------- 228 
TgR45        ------------FTPASK-RSLQYSVTKILISLHSN---------------GIAHLDLTTENILDHAYTEELPSSMTTQSGFDVSAHGSDGSSTTPSAKL 242 
CpR45        ------------LTDKEK-KELQYKLALALNNLHSK---------------GLCHLDFTPENILIGPNG------------------------------- 229 


Subdomain                 VII                                              VIII                             IX
                     179         193                                 194           210                  211           
                     |             |                                 |               |                  |
cAPKa        --------YIQVTDFGFAKRVKG---------------------------------RTWTLCGTPEYLAPEII------------------LSKGYNKAV 177 
Jnk1         --------TLKILDFGLARTAGTSF-------------------------------MMTPYVVTRYYRAPEVIL-----------------GM-GYKENV 181 
Flg          --------VMKIADFGLARDIHHI-------DYYKKTT-------------------NGR--LPVKWMAPEAL------------------FDRIYTHQS 204 
ERK1         --------DLKICDFGLARIADPEHDHTG---------------------------FLTEYVATRWYRAPEIML-----------------NSKGYTKSI 185 
PfPk5        --------ELKIADFGLARAFGIPVR------------------------------KYTHEVVTLWYRAPDVLM-----------------GSKKYSTTI 179 

                     410       420       430       440       450       460       470       480       490       500    
             ....|....|....|....|....|....|....|....|....|....|....|....|....|....|....|....|....|....|....|....|
MAL7P1.144   --------DLRLCDFAKSTPMYSNKLRHL--KESEDS---------------YKFESYETHVAKSAYTPPE---------------------CWEIYWRY 331 
PrR45-4      --------DLRLCDFAKSTPMYSNKLRHL--KESENS---------------YKFESYETHVAKSAYTPPE---------------------CWEIYWRY 330 
PF10_0160    --------EIRLCDLAKSTPIYTNNLRHL--KNMNGT---------------YLFESCVPTIGKIRSMPPE---------------------CWEISRKY 287 
PgR45        --------EMRLCDLSKSTPIYTYNLRHI--KDVNRL---------------NLFESCEPTIAKGAYMPPE---------------------CWKIYWKY 285 
PkR45        --------EMRLCDLSKSTPIYTYNLRHV--KDVNRL---------------YLFESCEPTIAKGAYMPPE---------------------CWKIYWKY 286 
PvR45        --------EMRLCDLSKSTPIYTYNLRHI--KDVNRL---------------YLFESCEPTIAKGAYMPPE---------------------CWKIYWKY 286 
PrR45-2      --------ELRFCDLSKSTPIYTYNLRHI--KDMNRL---------------YLFESCEPTIAKGAYMPPE---------------------CWKIYWKY 285 
PbR45        --------EMRLCDLSKSTPIYTYNLRHI--KDVNRL---------------YLFESCEPTIAK-AYMPPE---------------------CWKIYWKY 287 
PyR45        --------EMRLCDLSKSTPIYTYNLRHI--KDVNRL---------------YLFESCEPTIAKGAYMPPE---------------------CWKIYWKY 288 
MAL13P1.109  --------EMRLCDLSQSTPIYTNKLRHK--EKLNSI---------------KPFESFEPCIGKIEYIPPE---------------------CWKIVWKY 288 
PFC0060c     --------QMLFCDFAKSTPLYSFKLRHL--KHFEGL---------------YSFESCEPSIGKIEYMPPE---------------------CWNLLRKY 285 
PrR45-5      --------QMLFCDFAKSTPLYSFKLRHL--KHFEGL---------------YSFESCEPSIGKIEYMPPE---------------------CWNLLRKY 279 
PFI0105c     --------GMRLCDFGKSTPVYSYNLRHI--KNENGL---------------CYFESCIPSIGKVAYIPPE---------------------CWEIKKLH 284 
PF14_0733+4  --------EVRLCDLGKSTPVYTTSLRHL--DDSLDL---------------AIFESCVPCVGKEAYMPPE---------------------CMKLYKEY 291 
PFE0045c     --------ELRLCDLAKSTPIYTRKLRHV--QETKGL---------------CLFESCVPTVGKSAYMPPE---------------------CWKIYKRH 285 
PFD1165w     --------ELKLCDFANSAPIYTYNNRHLK-GN-KRL---------------RYYESYQPCISKVPLLPPE---------------------CWNIVRIH 286 
PF10_0380    --------EFRLCDLAKSAPMYTYNLRHIK-GDEKKS---------------FLFQSYQPCIGKLTCMPKE---------------------CWDIVKEY 327 
PrR45-1      --------EFRLCDLAKSAPMYTYNLRHIK-GDEKKS---------------FLFQSYQPCIGKLTCMPKE---------------------CWDIVKEY 329 
PFD1175w     --------ELRLCDLAKCAPMYTHNLRHIK-GNGNDL---------------YSFQSCQPCVGKIPCIPKE---------------------CWDIIREH 331 
PFA0130c     --------DIRFCDFGKSAPVYTTKLRHT--KEMNKM---------------ILFESCQPNIGKNPNTPPE---------------------CWDLFLKY 317 
PFL0040c     --------DIRLCDFAKSTPLYSDKLRHIDKKKKKKV---------------YLFESCVPTIGKREYTPIE---------------------CWRIRKKL 286 
PF11_0510    --------EFLLCDFCKSTPIYTTTLRHV--KEMNHI---------------CLFESCVPKIGKISYAPPE---------------------CIQLRKIH 295 
PFI0095c     --------EFRICDFGKSTPLYTNKIRHI--KKVNTM---------------CSFESCSPYVGKVPFIPPE---------------------CLKLYKLF 286 
MAL7P1.175   --------EIRLCDFGKATPMYTYNLRHI--NNINCI---------------HSFESCAPCVGKIRFIPPE---------------------CXELIKKQ 271 
PFI0110c     --------KMRLCDFAKSTPLYTYYLRHR--KNPNGL---------------CLFQSCCPTVGKPKYEPPE---------------------CVDLWRKL 284 
PrR45-3      --------KMRLCDFAKSTPLYTYYLRHR--KNPNGL---------------CLFQSCCPTVGKPKYEPPE---------------------CIDLWRKL 284 
PFI0115c     --------DMRLCDFSKSTPKYSYYLRHT--KKMKNL---------------CLFESCIPSVGKTRYIPPE---------------------CWDLVKIY 285 
PFI0120c     --------DMRLCDFAKCTPMYSYYLRHT--KKMDSL---------------CLFQSCITTIGKYMYVPPE---------------------CWDVEKRY 285 
PrR45-6      --------EMRICDFAKCTPRYTYNLRHL--RNPNGL---------------CLFESCIPTIGKIEYIPPE---------------------CLEIEKIY 287 
PFI0125c     --------EMRICDFAKCTPRYTYNLRHI--RNPNGL---------------CLFESCIPTIGKIEYIPPE---------------------CCEIEKIY 345 
PFI0100c     --------EMRLCDFGKTTPLYVLNNIDE--HNKGHL---------------QRFRSYIPYVGKTKYAPPE---------------------CWNLKKKY 282 
TgR45        SGGSVRRVQLKVCDLAKAAPLFNHSPFRLPPCILKAHFGDSGQETPVSSTAAQPFLSCEPTVAKGPYMPPE---------------------CWRIVYIL 321 
CpR45        ---------INICDFAKSTPIITKNPRHTHYSRKSPKNHLS-----------ENFNSNN-TSFNFINPPPEAYCEFESCEPTVGKGAYMPPECWKIFWRL 308 


Subdomain              IX                                                            X
                                 240                                       241                260
                                   |                                       |                    |
cAPKa        DWWALGVLIYEMAA--GYPPFFA---------------------------------------DQPIQIYEKIVSG-KV-RFPSH---------------- 218 
Jnk1         DLWSVGCIMGEMVC--HKILFPGRDYI-----------------------------------DQWNKVIEQLGTPCPE-FMK--KLQPTVRTYVE-NRPK 240 
Flg          DVWSFGVLLWEIFT-LGGSPYPG---------------------------------------VPVEELFKLLKEGHRMDKPSN----------------- 247 
ERK1         DIWSVGCILAEMLS--NRPIFPGKHYL-----------------------------------DQLNHILGILGSPSQE-DLN-CIINMKARNYLQSLPSK 246 
PfPk5        DIWSVGCIFAEMVN--GTPLFPGVSEADQLMR------------------------------IFRILGTPNSKNWPNVTELPKYDPNFTVYE---PLPWE 244 

                     510       520       530       540       550       560       570       580       590       600    
             ....|....|....|....|....|....|....|....|....|....|....|....|....|....|....|....|....|....|....|....|
MAL7P1.144   YELKIKEPLEYLKL---------------------------------ITNQEERKQFYFDVACADKFMLGVLFIWIWTSGNLWVCSDPLQDDYFHCLMKS 398 
PrR45-4      YALKIKEPLEYLKL---------------------------------ITNQEERKQFYFDVACADKFMLGVLFIWIWTSGNLWNCSDPLQDDYFYCLMKS 397 
PF10_0160    IRMKICEPLEQLSP---------------------------------ITDLDERAPFYFDVTSADKFMLGVLFIWIWNNDYLWKSSDIEQDIDFLRVSEC 354 
PgR45        DTMKIKNPLKDLKS---------------------------------ITDQDKRKQFYFDVSSADNFMLGVFFFWIWTNGNLWKCSDPLQDEDFFYFVKC 352 
PkR45        DTMKIKNPLRDLKN---------------------------------ITDQEKRKQFYFDVSSADKFMLGVFFFWIWTNGNLWKCSDPLQDEDFFYFVKC 353 
PvR45        DTMKIKNPLKDLKN---------------------------------ITDQEKRKQFYFDVSSADKFMLGVFFFWIWTNGNLWKCSDPLQDEDFFYFVKC 353 
PrR45-2      DTMKIKNPLKDLKH---------------------------------ITDQEKRKQFYFDVSNADKFMLGVFFFWIWTNGNLWKCSDPLQDEDFFYFVKC 352 
PbR45        DTMKIKNPLRDLKS---------------------------------ITDQEKRKQFYFDVANADKFMLGVFFFWIWTNGNLWKCSDPLQDEDFFYFVKC 354 
PyR45        DTMKIKNPLRDLKS---------------------------------ITDQEKRKQFYFDVANADKFMLGVFFFWIWTNGNLWKCSDPLQDEDFFYFVKC 355 
MAL13P1.109  KMNNIKNPIEYLKN---------------------------------ISNQEERKKYYYDVSCADKYMLGIFFIWMWNNGFIWKCSDPIQDKIFEIFMKS 355 
PFC0060c     KKMKIKDPMRNLNY---------------------------------ITNQDKRKDFYYDVSNADKYMLGVFFIWVWNNGYLWSRSDPEQDHVFYELSQA 352 
PrR45-5      KKMKIKDPMRNLNF---------------------------------ITDQDKRKDFYYDVSNADKYMLGVFFIWVWNNGYLWSRSDPEQDHVFYELSQA 346 
PFI0105c     KTEKIRNPYTSLRN---------------------------------ITDQGERKRFYFDVLAADKFMVATFFIWLWNEGHLWDKATASQDEIFFKIVEK 351 
PF14_0733+4  RKMKISSPFDYANS---------------------------------VRDRRERRKWYFDVLTAEKYMLGIFFMWIWNEGHLWDCSDPSKDEIFNEINEC 358 
PFE0045c     RILNIKNPLQHLNA---------------------------------ITVQDERKKHYFNVTCADKYMLGILFIWIWNDGHIWHCSDASTDENFYQFEKC 352 
PFD1165w     EKLKINDPYDYLKS---------------------------------ITNQEERKSFYFNVSNADKFMLGIFFIWVWNNNYIWEKADSFNDRKFRNFVKC 353 
PF10_0380    MRLKIKNPLEYLKS---------------------------------IKNQEERKKFYFDVLSADKYMLGILYIWIWNNNYIWKRADPSKDKIFSHLLNY 394 
PrR45-1      IRLKIKNPLEYLKS---------------------------------IKNQEERKKFYFDVLCADKYMLGILYIWIWNNNYIWKRADPSKDKIFNHLLNY 396 
PFD1175w     IKLKIDNPFEHLST---------------------------------ITDQEERKKYYFDVHCVDKYMLGIFYIWIWNLNYIWKRADPPNDRTFNNFLKY 398 
PFA0130c     ESLNVEDPLEYLKT---------------------------------IKDPEERKIYYFDVRSADKYMLGIFFIFLWNDGYLWDSAE-MEDDDYSKFVKS 383 
PFL0040c     REKNITDPFEHVKT---------------------------------ISMQRFRKEYYFNVSHADYFMLGVLFIWIWNCGHMWKTSFPSESVNFTTFLEN 353 
PF11_0510    EKMDIKNPLSDLNY---------------------------------IKDIEERRKYYFDVTSADIYMLGVLFLRIWNSKPLWLIANIEEDLNFSKIFEA 362 
PFI0095c     RERSIREPLKYLHS---------------------------------IRDTEERRKYYFDVTSADKYMLGILFIWIWNDHHLWECSDPLTDKNYMKFEKN 353 
MAL7P1.175   EELDITYPLEYLKS---------------------------------ITDQEERKTFYFNVSSVDKYMLGIVFIWIWNYNFLWKRSDPSYDLQYLKFEQF 338 
PFI0110c     KEMKINNALMHLRN---------------------------------IEDQEERKKYYFDVECVDKYMLGIMLIWIWDYKYLWNKADSLEDNDFILFKEN 351 
PrR45-3      KEKKISNALMHLRN---------------------------------IEDHEDRKSYYFDVACVDKYMLGIMLIWIWDYKYLWNKADSLEDNDFILFKEN 351 
PFI0115c     IKENVDYPFEYLKN---------------------------------MYDDEERKQYYFDVSCVDKYMLGILFIAIWNNGYLWYKSDPLQDKDYLKYSKS 352 
PFI0120c     VEVGTKFPFEYLQE---------------------------------ITDEEERKKYYFDVSCADKYMLGIVFIAIWNNSYLWYISDPIIDINYLKYKKC 352 
PrR45-6      KEHKVKEPFSYLKT---------------------------------IIDQEERKKYYFDVTSADNYMLGILFILIWVYHFFWDKADASVDKEYAEFARV 354 
PFI0125c     KELKITKPFSYLKT---------------------------------IIDQDERKKNYFDVTSADNYMLGILFILIWVYHFFWNKADASVDKEYAEFARV 412 
PFI0100c     KELGIENPLVYLKT---------------------------------LKDYEYKDTLYFDVLAADIYMLGILFIWISSNRYLWGNFDMSQNSNFKKFVNS 349 
TgR45        RALGITAPFAQVGEPLLTTGRPPPPLYLKDPSQSTAQLFGAVHDPIDVSAGVDAAELFFDVRKADVYMLGVLLFWIWAEGAIWTCSDPRQDAQYNDLLQC 421 
CpR45        EENKIQFPLEELMDIDTYNRRSNYSYFNEFSRIHNKSP---------IKCQ-DRSMFYFNTRIADIYMLGIIMFWIWSDGGIWKYSDTRQDHRYQNLVFS 398 


Subdomain                                          XI
                                 261                               297
                                 |                                   |
cAPKa        --------------------FSSDLKDLLRNLLQVDLTKRFGNLKNGVNDIKNHKWF 255 
Jnk1         YAGYSFEKLF(14)------KASQARDLLSKMLVIDASKRISVDEALQ-----HPYI 286 
Flg          --------------------CTNELYMMMRDCWHAVPSQRPTFKQL-------VEDL 277 
ERK1         TKVAWAKLFPK---------SDSKALDLLDRMLTFNPNKRITVEEALA-----HPYL 289 
PfPk5        SFLKGL---------------DESGIDLLSKMLKLDPNQRITAKQALE-----HAYF 281 

                     610       620       630       640       650       660       670          
             ....|....|....|....|....|....|....|....|....|....|....|....|....|....|....|.
MAL7P1.144   DMNFNNFPCSQN--------WPHGLKHIIKQLLHMKY--RKDLNLNILGI---HPWWYKKK                446 
PrR45-4      DMNFNNFPCSQN--------WPHGLKHIIRQLLHMKY--RKNLNLNILGI---HPWWYKKK                445 
PF10_0160    DMDIDVFELTRT--------WPYELKKIIQKLLQTEG--RKNLNLHELCA---HPWWFSKM                402 
PgR45        DMDFDKFELTKK--------WPNELKNIIKQLLHIDY--RKKLNLKDLSA---HPWWSCKL                400 
PkR45        DMNFDKFELTRK--------WPSDLKNIIKQLLHAEH--RKKLNLKDLSM---HPWWSCKLQ               402 
PvR45        DMNFDKFELTRK--------WPTDLKNIIKQLLHVEH--RKKLNLKDLSM---HPWWSCKLQ               402 
PrR45-2      DMNFDNFELTKN--------WPNELKDIIKQLLHVEQ--RKKLNLKDLSA---HPWWSFKL                400 
PbR45        DMNFDKFELTRK--------WPSELKSIIKGLLHAET--RKKLNLKDMI----HPWWSCKL                402 
PyR45        DMNFDKFELTRK--------WPSELKSIIKGLLHAET--RKKLNLKDMI----HPWWSCKL                403 
MAL13P1.109  NMDLNKFIMTKS--------WPHELNNLINKLLHMEH--RKTVKLSDLSR---HPWWSSKN                403 
PFC0060c     DMNFNMLERTKK--------WPDDFKFILKKLLHMEH--RRNLDLKDLCK---HPWFTSKK                400 
PrR45-5      DMNFNMLERTKK--------WPDDFKFILKKLLHMEH--RRNLDLKDLCK---HPWFTSKK                394 
PFI0105c     GMNLKALELTRK--------WPRELKSIIKNLISLES--RKSFNLKDLID---HPWFTNK                 398 
PF14_0733+4  EMDLDKCDLTDN--------WPEGLKAMIKRLLNFES--RKELNLKDIYD---DPWWSTIM                406 
PFE0045c     DMSLDVFQLTST--------WPSGLKNILNELLHIEK--RKMLVLRNLLS---YPWFTKENDFSL            404 
PFD1165w     GMDLYNYELTYN--------WPDDLKDIINQLLPLEN--RAQLSLKELCK---HPWWSN                  399 
PF10_0380    NMDINSIKLAED--------WPKGLKNIINKLLDLES--RMKINLDDLVK---HPWWFYDE                442 
PrR45-1      NMDINSIQLAEQ--------WPKGLKNIINKLLDLES--RMKINLDDLVK---HPWWFYDE                444 
PFD1175w     NLNINVFQLAKQ--------WPKGLKDIINKLLSLES--RMKTDLNELTE---HPWWINED                446 
PFA0130c     DMNFDSFELTKS--------WPEGLKVILKQLLDENN--RKNLNFNDLVI---HPWWSYKN                431 
PFL0040c     NMNLSCYPSTKS--------WPSDFKFIVKELMNEEC--RKKLNLKNLMT---HPWFNET                 400 
PF11_0510    DMNFDKFVIAKN--------WPKEFKKIIQQLLHMTS--RKNLSLKELSK---NPWWKE                  408 
PFI0095c     NMSLDTFRVTKS--------WPEDIKIMIKNLIDIKY--RKNIKLKNLIK---HPWWFKN                 400 
MAL7P1.175   DMILDFFKKTKR--------WPKELKNIIKQLLHMDY--RKNLNLNDLSK---NPWWSSNI                386 
PFI0110c     NMNLDIFPTTQT--------WPEELKYIISQLLVLET--RKNLQFKDLIN---HPWFSCNE                399 
PrR45-3      NMNLDIFPTTQS--------WPEELKHIISQLLVLET--RKNLRFKDLIN---HPWFSCKE                399 
PFI0115c     NMNFNKFWTTFF--------WPKELKIILRQLLDLEC--RKNLNLNDLIS---HPWFSKK                 399 
PFI0120c     NMNFNKIWTTLF--------WPKKLKRILRQLLDLDR--RKNLNLNDLIN---DPWFTR                  398 
PrR45-6      NMDFHEVEKTYY--------WPDGIKFIIQQLLYFEY--RKDLDLNDLIN---HPWFTTEEYWFLKLFSFSLTYFK 417 
PFI0125c     NMDFHKVEKTYH--------WPDGIKFIIQQLLHFES--RKNLDLNDLIN---HPWFTREKCWFLKLFSFDLRYFK 475 
PFI0100c     DMNFDLFPLTRE--------WPEGLKYIIRKLLDYES--RKSLDLNELIE---HPWWSTDL                397 
TgR45        GMEFSIFIDCDG--------WPPELRHLLKGALDPDPTRRVTLPEILQ-----HPWWTCSLAATGQEAALTS     480 
CpR45        DINFDVFRECRG--------WNKQLKSLLKKMLEPDPNKRITMSEILS-----DPWWKCPLDE              448 
