# Supplementary material for: A new Apicomplexa-specific protein kinase family : multiple members in Plasmodium falciparum, all with an export signature
Source: BMC Genomics. 2005 Mar 7;6:30. doi: 10.1186/1471-2164-6-30 (PMC1079819; doi:10.1186/1471-2164-6-30)
Supplement: Additional File 2 — Multiple sequence alignment of the N-terminal region of R45-like proteins in P. falciparum and its orthologs in P. reichenowi. [file 1471-2164-6-30-S2.rtf]

Additional File 2


Multiple sequence alignment of the N-terminal region of R45-like proteins in P. falciparum and its orthologs in P. reichenowi. 
Sequences are given with an aligned exon 1 / 2 boundary. The exon boundary is indicated by a " / ". The predicted signal / anchor sequence (trans-membrane domain for PFI0100c) is highlighted in light blue. The Pexel motif is underlined and the three conserved positions are highlighted by colours. Sequences from T. gondii and C. parvum are indicated below.


                      10        20        30        40        50        60        70        80        90       100                  
             ....|....|....|....|....|....|....|....|....|....|....|....|....|....|....|....|....|....|....|....|
MAL7P1.144   -------------------------------------------MYYSVNEWKINKFIHSIFPKKSILIIFLILYL/----------RNLTKRKYNILRGL 47  
PrR45-4      -------------------------------------------MYYSVNEWKINKFIHSVFPKKSILIIFLILYL/----------RKLTKRKYNILRGL 47  
PF10_0160    ---------MTLINRSYVLFGEKLKINYINIKKKKKKFLSDYSCRKKGNCKYGMSTVHTLYFIWFVLGTLYFFLS/-IFDKDGMKSVSDLVIIQRYNRCL 90  
MAL13P1.109  -------------------------------------------MKKKENTQKLLNFVYYKIYLTFIVGLLYIFLL/NILINHGGSKNDVRFTNIRCVRIF 57  
PFC0060c     ---------MFNFILYDLNLTPKKNLIYKSKYIKEYTYNYFNYVISNKEPKYFLCEFLTHRCTVTFLGILYFILI/FSGTFNHTSVSSYQIKEKAYCRFL 91  
PFI0105c     ---------------------------------------------------------------MKFIGVLFLFLL/-NVIIYETNIILNDKIFERYNRSL 36  
PF14_0733+4  MIPHRKWYSILCVEYFIYSNSNLKKNNCIYSLNSEKNAKKDKNEKAKWRPKELYCNLFLLIFVILTIGCTYVKIE/-NLSSLSKGSILQDKIISRYSRGL 99  
PFE0045c     -------------MLGFNFFCSFLNEYTLKENIIRRNDRVCASYMDKKRKLKHCSLSRPFNIQSFVSVLIFSLLV/-IFVSGDKIFIDSLDIKSSHCRKL 86  
PFD1165w     -----------------------------------------MKKKTREEIIIFLKWVSCKKYIFSVFVFLCCFLM/SFNIFEPRIEIIELNWHVRYYRHL 59  
PF10_0380    --------MGNFCKYNYMETNRLLYMKCFKCNNSPMYTISKKDEYKKKESWNFVNSLLSRNLILFTLTFLFLNIL/-NCVNYNQYDYPSQHIINNSIRHL 91  
PrR45-1      ---------------------------------------------------------------------------------------------------- 1   
PFD1175w     --------MNYFSKYKVIESNRLLYTSNVKYDNYFININKKNQVKNKNESYSFIKLLFRKCIIFFIIYFLFIIPL/-NEVIYNKYDYSSKSIQHYCIRNL 91  
PFA0130c     -----------------------------------------MLNMIQKKKNLFLRSILIKFIIISFLGILYLFFY/-NVEVLEDKETNNLELIVNESRYL 58  
PFL0040c     --------------------------------------------------MYILRNMFCIKFMLYFLWLLYLLFL/-NIEFIKFKTFQSLVSYDRPSKCL 49  
PF11_0510    --------------------------------------------------------MIYIKLRLYIFWFLFLILL/-NLTLTDRKIFEYIRLIDIYFRIL 43  
PFI0095c     ------------------------------------------------------------MLILMRMLIFASLII/-VFINVENKAIGNLNLVNVNVRSL 39  
MAL7P1.175   ---------------------------------------------------------------MHNIFNNICSLLMPILVVMDKWTNKPLNIIYEHSRNL 37  
PFI0110c     ---------------------------------MEIKCPLKYIKNKYNNKYIGLFLNLKGFVMYLLVISLYFFFL/-FLTEIQNKCIAKGCVNIICPRKL 66  
PrR45-3      ---------------------------------MEIRCPLKYIKNKCNNKYIGLFLNLKGFVIYLLVISLYFFFL/-FLTEIQNKCIAKGYVNIICPRKL 66  
PFI0115c     ---------------------------------MELICVFFMVQKNNIYIYILILYYMDVRVCIYKNILYVMHID/-IIRVIQINNDYNSFTHFNNSRYL 66  
PFI0120c     ------------------------------------------------------------------MEFLYFIFQ/-VLSLIRINQNTVFYRSLKKCRYL 33  
PFI0125c     --------------------------------------------------------------MLILYFYLIVSEN/--------NIFSELYTYNITLRHL 30  
PrR45-6      ---------------------------------------------------------------------------------------------------- 1   
PFI0100c     --------MSFYNCSDYNFNKDQLCNKNVYSEIKIIAPFHKNVEESKKINYNLKTWLSRCLIIAQTIILVYTYLF/-HLNVLSCNINSDSISCKGIIRNL 91  
                                                                                                                  1   
TgR45        ARCRHLATFDLQTLFLCCGFVGSRIAAVLRTSSPKSPAPPQAPQNRGSSPDDNKVRHRPTLTPFFAEQGGRTEDTHYGQDHRGDTNNSALITARGEQGRQ 100 
CpR45        MKNFSKEETRKPFTNRQVIDIKSRHDQSGRHLKTNGEFGYESGIWSKFVGLMKNSLNISILNRPSNQLNKVMDNTSSKNHLKEIIMKKSTEKFNDKSAKL 100 


                     110       120       130       140       150       160       170       180       190       200         
             ....|....|....|....|....|....|....|....|....|....|....|....|....|....|....|....|....|....|....|....|
MAL7P1.144   LEFEQILGRCPEHKAKKGHKKMKFRKSKNEKKNEQQDDLLKNKEDDLLKNKEGDLLKNKEGDLLKNK-GDLLKNEEGDLLKNEEGDLLKNKGDLIKNKEG 146 
PrR45-4      LELEQILGRCPEHKSKKGHKKMKFRKSKNEKKNEQQDDLIKNKEGDLIKNK-GDLIKNK-GDLIKNKEGDFLKNK-GDLIKNKEGNLIKNKGDLIKNKEG 144 
PF10_0160    AEFGSVRNIFNWSVFTHVFEKMKNIDLLFEDDEEDTYKNEKTILSKINVKEKWNKYRPRTNKIKRALTKIELKDNLNRFIKCLNKDRLEIVNDYNSDRSY 190 
MAL13P1.109  SENIKNINEISKKIYLYNIKNEKDDIICRDSLDNINEINNKINYTSVKGEDIIILKEGKYKNGCSDINFLDRKDVNKNDDQSFRNYHKTNNNKDENVNMK 157 
PFC0060c     SEHDYVKDCSDENMLSRIFKKILLRKKEHHEKFEDEQEDGRFLFSNSSIKDKWSKNKRISSIGDDIKSEFWSKENLMYNIKLLKNEKIEVNDYDTSDDDC 191 
PFI0105c     SVFIRDNNKDEEGTKELLLRKIEKEEEVYDLKDVRNDYMNDSIKDNNNHSTNDYLKGTTTDSTKFHIYDNIKCTLQYDKNDSTKNVIYDSTNTTVIEDSN 136 
PF14_0733+4  CEIESVNNGSNRSVFTRIFNRIRGEEKKRNCEEPDKNIMDYLLKNDLNLKDKKQKSTNRSTIDIPTLDIEENGSPLIDITDIPFIESSKKSNEEEHEETL 199 
PFE0045c     NESVNVIVNENFNSEDNKLKGISKIDENRLNHEVLYNNRKISIKKHENKDESLENSNKNFELNGVDLYHGNVELKNIKNLDDNDKMDLKHEYNNENVGDT 186 
PFD1165w     TEVWDNNNNNDEGEQNLKDKYDTYDEKEKVKKKYVEDEFNTKEKVKKKHRDDKYNKKEKSKKKHRDDKYNKKEKSKKKYVEDKFNKKNKHKENDVSLKKD 159 
PF10_0380    SDYSSSNDNLSDEQEYFTTSDDSEEEKKHNEKINKKRKKCKKYKKRRGKLREKKKGKSNLYHNGKNKKNVSNCKKHNINLKNNLNNNNDLLNNNYNISND 191 
PrR45-1      ----------------------------------------------RGNLGEKEEEKSNIYYNGKNKKNQSNCKKHNINLKNNLNNNNDLLNNNYNICNN 54  
PFD1175w     SECFRGKSALNDDSESCRCSNLLKEKKKTEENSEEIRKYLSDKTKKRNSLYKYKLKGSKGKSVKNIVGNKEGSIHENTDLYSDSDVEYDYGNLFYDCYYT 191 
PFA0130c     AESRKDLEKKGDKPNNINVDNFGVRGSKNKCAINSEERRGNNCEGISKLFVGCTKGLKKFWNKISCDPLCIEKNDSNSDNNYNNDNNIQNNTCSVNNDLL 158 
PFL0040c     SENSKHHVNSDNNKGNKLFGQKQFGNINKCDVKDDELKISKDNTSKKKKICFKKEKRSNEEEYNNLEKESVEGTCNLLNILNVEKTKVFDNYESTYKHGE 149 
PF11_0510    YEYNGINKLCGGKSCNKIFDQRILGEEECIYKGDDKKKKKTKLIDMINLCKIWNKIKKVIYKDENILKSGSNLNIKENKKFIYKLNGENTGINDNQQYID 143 
PFI0095c     CAKNEKFVAKKKKKKSIFTKIRVKGSKTLEGFLCDTLNGIIFKSTKEKSDNESIDKDSDESISYMEEIDVNNEEDNNVNLCRYNNMIDNNSINVLENVNN 139 
MAL7P1.175   SEYEKLLNTSNIPFIRSIFTNKGIKKKSNNWLLNKKTNMCLFGRLNKNNCEDNFFEDRTNDTDWGGDKHRFCKLNIMNKENEYINGIIDNNWDSIKYMNY 137 
PFI0110c     AESNIANERSSYSFFNRLFKKKKTKRQKRKIEDEKIEKEETEEEKSKIKKESKDITNENSKYEKNQNNLRCQDNNNIVSRIYDNGMNKKELLFTEYNKKD 166 
PrR45-3      AENNIANDRSSYSFFNRLFRKKKTKRQKRKIDDKKIEKEETEEEKSKIKKESKDIRNENSKYEKNQNNLRCQDNNKIVSRIYDNGMNKKELLFPEYNKKN 166 
PFI0115c     SEYSSDIETLNNSTFHNTCIKDNIKSDKYLVNINDEKNKLNKEINNIKNYENNLYCNHNCEKFNHDTYVNKLFIKDEIYYNEPYFKENIIENVNYVLEDL 166 
PFI0120c     SEIIIENVTSTKLYSDDCYDKNGVINDNNYVLKNTYDEYESYKRIKKIKQCKNILKENKLSESILPESTLPESTLPESTLAESTLAESTLAESTLAESEF 133 
PFI0125c     NEYSLEDEKYGENFFSRIVNKIHKGKKSKDAITKYIKEIIYKNKLCKKINFCKDFSSDEKDDEITNVRKEEKNIKEIKNCSFLPNYISDNFEKGISYKNK 130 
PrR45-6      ---------------------------------------------------------------------------------------------------- 1   
PFI0100c     SEPCKVNEKSHETFIDRVFYGTKKKKDGSNKNKK                                                                   125 
                                                                                                                  1   
TgR45        LRRSRRSGGTAAHVVMEHDGDRQHAQPSNDSRGPGVEEIRVLKAWTHADTAEVEGRSADCGNVLRDSAALQAALRSPEQR                     180 
CpR45        GIGLREQKEVNIGIKSKGEKSPVSPKSTKISPSLSPVVSFPYSTSSPSSSSSSSFSSPSSLNENIGISFAEQILNFNSSEYSKVINGQAPVTPTTLDGSS 200 


                     210       220       230       240       250       260       270       280       290       300         
             ....|....|....|....|....|....|....|....|....|....|....|....|....|....|....|....|....|....|....|....|
MAL7P1.144   DLLKSKEGDLIKNKEGDLIKNKEGDLLKSKEGDLIKNKEGDLLKSKEGDLIKNKEGDLIKNKEDVLLNKGYNILQNKNDNLLQNEYYNLLQNEQDDNQLK 246 
PrR45-4      DFLKNKEGDLIKNK-GDLIKNKEGDFLK------------------------NKEDVLLKSIEDVLLNKGYNILQNKDDNLLQNEYYKLLQNEQDDNQLK 219 
PF10_0160    DDIIHEDENNINNLNSKY                                                                                   208 
MAL13P1.109  SY                                                                                                   159 
PFC0060c     DDNKISKYPKILKERDNSKP                                                                                 211 
PFI0105c     GKKNNDEGDYSLCNKKKKKRRKKKIEMKDNNNNNNSINNNNLYDEKTENVQIEKVVNRPPYVDIAKSLNEDDNVSNNSYDNFDMKTNIISDLEQEIKNKC 236 
PF14_0733+4  DEKILKCRSRLAKAATSKT                                                                                  218 
PFE0045c     NNLEEEIKDKT                                                                                          197 
PFD1165w     QCKKSLSIREDCNNKNSKSYNEKEICDNENCVNLEDIKIYDNTTWKYKIKKLITCSYNSDKDAKV                                    224 
PF10_0380    CYICEQMTSQNKTKVPYGYYEEIDYIDDDDDDDDDSFRNKISNEKIELVNGNSKDKGIYYKNNLGNEKKNDYRTEDQTHLGIELIEEEFKGRNGNLDEEN 291 
PrR45-1      CYNCEQMISQNEAYVPYGYYEEIDYIEDDD-----SFCNKISKEKIELVNGNSKDKGIYYKNYLGNKEENDYRTKDQTHLGIELIEEEFNGRNGDLDDEN 149 
PFD1175w     EGLIDQGEENEEEKFYEAREDFEGEEKNKCKIKESEKSFKKENDCKVENVENINYER                                            248 
PFA0130c     KNEFEIKDVNVKEVDDDIISRDMNKNCVNEINNMNKEKDSKI                                                           200 
PFL0040c     NNDIICMSNLKEDESKENY                                                                                  168 
PF11_0510    ELKDNIHSKN                                                                                           153 
PFI0095c     PPKV                                                                                                 143 
MAL7P1.175   AHNNESCSDSNN                                                                                         149 
PFI0110c     DSYIYMKVIDITPNKNNENLNANVDAIVPCVSELEKRLNSENKI                                                         210 
PrR45-3      DSYIYMKDIDIATNKNNENLNANVDDVVPCVSELEKRLNSENII                                                         210 
PFI0115c     EEFSPNNNP                                                                                            175 
PFI0120c     SKYKLSNDTINENLESHLYLNSSPSTSHSMYYHDSKCNENINESNNATNELMEKSVSIKYP                                        194 
PFI0125c     LEEEYMSVEN                                                                                           140 
PrR45-6      -----MSVEN                                                                                           5   
PFI0100c                                                                                                          125 
                                                                                                                  1   
TgR45                                                                                                             180 
CpR45        TTTSPTFTNLRFSSPSNNNGSAKYNETPNSKEFDSSTLKEEYFSNKKNHAVDFSADDNHLNYIMERINQSGLVESNDKSVAKLDIPLLNQYISQDLIAEV 300 


                     310       320       330       340       350       360       370       380       390       400         
             ....|....|....|....|....|....|....|....|....|....|....|....|....|....|....|....|....|....|....|....|
MAL7P1.144   GNTLITT-------------------KKEDKGCMKKTHENKAECEKNEDKNCMKKTHENKAECEKNEDKNCMKKTHGNKAEDEKNEDILLMSPTKGNNLW 327 
PrR45-4      GNNVITTKKEDKSCMKKTHENKAENEKKEDKSCMKKTHGNKAEDKKKEDKNCMKKTHGNKAEDEKKEDKSCMKKIHGNKAEDEKNEDILLMSPTKGNNLW 319 
PF10_0160                                                                                                         208 
MAL13P1.109                                                                                                       159 
PFC0060c                                                                                                          211 
PFI0105c                                                                                                          236 
PF14_0733+4                                                                                                       218 
PFE0045c                                                                                                          197 
PFD1165w                                                                                                          224 
PF10_0380    IDYNKERIRENIYHGLEKIEEEIKERMEELIDDGREIENEENREYNNNDIKKRRFDGDQENITSSFERTQKSVKYDSDESEELSEAESNGGIPEQNGYDN 391 
PrR45-1      IDYNKERIRENIYHGLEEIEEEIKERMEEIIDDGREIENEENIDYDNNDIKKRRFHGDQENITSLFERTQKSVKYNSDESEELSEAESNGGIPEQISYGN 249 
PFD1175w                                                                                                          248 
PFA0130c                                                                                                          200 
PFL0040c                                                                                                          168 
PF11_0510                                                                                                         153 
PFI0095c                                                                                                          143 
MAL7P1.175                                                                                                        149 
PFI0110c                                                                                                          210 
PrR45-3                                                                                                           210 
PFI0115c                                                                                                          175 
PFI0120c                                                                                                          194 
PFI0125c                                                                                                          140 
PrR45-6                                                                                                           5   
PFI0100c                                                                                                          125 
                                                                                                                  1   
TgR45                                                                                                             180 
CpR45        GSCLEENNKRISSNKYTLLGRGLSNLPKHSSTVSYSPSSLSSGKNDKV 									348 


                     410       420       430       440       450       460       470       480       
             ....|....|....|....|....|....|....|....|....|....|....|....|....|....|....|....|.
MAL7P1.144   TRLKKGFSRGMCMNFLLNDNNEKKLSTLYVTNMLKNQLNSYYGSKNSNDKKLEKSDNEGGEEKYDNSNKEQNM         400 
PrR45-4      TRLKKGFSRGMCMNFLLNDNKEKKLSMLYVTNMLKKQLNSYYGSKNSNDKKLEKSDNQGGEEKYDNSNKQHNM         392 
PF10_0160                                                                                      208 
MAL13P1.109                                                                                    159 
PFC0060c                                                                                       211 
PFI0105c                                                                                       236 
PF14_0733+4                                                                                    218 
PFE0045c                                                                                       197 
PFD1165w                                                                                       224 
PF10_0380    EDVVSGSDENSDIEKNTEIEEANGYGSEDNSCATEEGSDSNLEENTEEGIEDGIIGLIQNIFQGSDKEDEEEKEKNNKKKN 472 
PrR45-1      EDVVSGSDENCDVEKNTEMEEANEYGSEDNSCATEEGSDTDLEENREEGIEDGIIGLIQNIFQGSDKEDEEEKEKNNKKKN 330 
PFD1175w                                                                                       248 
PFA0130c                                                                                       200 
PFL0040c                                                                                       168 
PF11_0510                                                                                      153 
PFI0095c                                                                                       143 
MAL7P1.175                                                                                     149 
PFI0110c                                                                                       210 
PrR45-3                                                                                        210 
PFI0115c                                                                                       175 
PFI0120c                                                                                       194 
PFI0125c                                                                                       140 
PrR45-6                                                                                        5   
PFI0100c                                                                                       125 
                                                                                               1   
TgR45                                                                                          180 
CpR45                                                                                          360 
                                                                                               1   
