# Supplementary material for: A new Apicomplexa-specific protein kinase family : multiple members in Plasmodium falciparum, all with an export signature
Source: BMC Genomics. 2005 Mar 7;6:30. doi: 10.1186/1471-2164-6-30 (PMC1079819; doi:10.1186/1471-2164-6-30)
Supplement: Additional File 3 — Multiple sequence alignment of the N-terminal region of R45-FIKK kinase orthologs from six Plasmodium species. [file 1471-2164-6-30-S3.rtf]

Additional File 3

Multiple sequence alignment of the N-terminal region of R45-FIKK kinase orthologs from six Plasmodium species. 
The orthologs that cluster together on a phylogenetic tree based on their kinase domain were aligned. Positions with identity in 4 out of 6 sequences were high-lighted with colour.


                  10        20        30        40        50        60        70        80        90       100                  
         ....|....|....|....|....|....|....|....|....|....|....|....|....|....|....|....|....|....|....|....|
PgR45    MIKTTEKIDYSKSETLPCDDNYLSLKSEKTINLDTEKCEILLSEKKKKVNEN--------FSNKCNVSKNKVISS----------------------NSL 70   
PkR45    MIESTGKVEYSKSKTIPCDGINDDDEEEASKYLPVKYEKTLDLENANQTSNAVDDMEKKCQADEMSNSAKEAKSKEIKCVNSGKEGNKVNVSKCEKVQTE 100  
PvR45    MIETTGKVEYSKSKTIPCDGSNDDDEEEANNYLPLKYEKTLDLEKGNELSGTQ-------QSGELASSPKGAKSK------------------GVKGDAA 75   
PrR45-2  -MDYPDDFKQRTNQKFNSTIMMKDNRDDIKN---VYRNISMDKDVSG-------------INNEMNVEEDNNVSFG------------------------ 59   
PbR45    MIDSADKIEYSKEKTLACNGGNKDYEQDE---SSLKYKKTLDMDTI--------------ENDFYNCMEKESICD------------------------- 58   
PyR45    MIDSGSEIEYSKEKTLSYNGDNEGCEQDE---FSLKYKKTLDIDTN--------------ENDVYNSMEKENICD------------------------- 58   

                 110       120       130       140       150       160       170       180       190       200         
         ....|....|....|....|....|....|....|....|....|....|....|....|....|....|....|....|....|....|....|....|
PgR45    YNDKIKDNIELCSIVNSTQTFIRNDFQYNNQMSNKEKKISRKSNTSKKGNDKNIEENEK-DFFSNST----------------MYYKN-TISTKYYDN-- 150  
PkR45    VEFSKTDTLDGVTMDTTSEGGIP--LSLITKVGVKKAYLGNSPEFSNQAGKGGLPESGD-HMEEDGDNMCMVRMDTELSSNGNMYNRN-VVSTKYYETMG 196  
PvR45    VEPPKMGTLDEGTMETTSGGGTP--FDLNVSMGMKKALHGDAPANSSQAGNGGLPQILD-HLEEETD---------------NMYSRK-VASTKYYG--- 153  
PrR45-2  ----NNSNLESYENMGNESYTEN--IMYRNKKEQDEDEDDIDEEDKNSNNNYNLNDHNN-TIEAERK-------------------KNDTFYEKNED--- 130  
PbR45    ----EKNKLEQFSVNKTCNVLKK--SVYTESGSKDSYYFQNIKDISKKNEDKNYKEVDDNDLSTNVN---------------NLYNKN-IIGTKYYD--- 133  
PyR45    ----EKKKLEQFSVNKTCNGLKK--SVYIESGNTDLSYFQNIKDISKKNEDINYKEVN--DLSINVN---------------NLYDKN-IIGTKYYD--- 131  

                 210       220       230       240       250       260       270       280       290       300         
         ....|....|....|....|....|....|....|....|....|....|....|....|....|....|....|....|....|....|....|....|
PgR45    --INEIDSKKNNDK------CFYVNESKNNDSLKNLKNNTISRNNNPIIEEKELLMNHIENKKKINNLLGNYTESSLSKCRISDINKSNSKNYN-NYSNN 241  
PkR45    GSGTGSSGKSRDNAESVYPKEFSSIERKEGEESCSPYGMDAGGMKN--NVERASLGALSVGTFATD-------CSNISKEEKEIFAKMIDDQTKKNIING 287  
PvR45    --TGSSGESRDNEESGYPKGFSSPVQGKKGEGASPYGVDADGVKHSMAEASKASKGGLPEDTYTTN-------CSNLAEGEKDMFAKMMEEQAKKSSING 244  
PrR45-2  -----YKNMLSN-----------QDIYNETENVNDNTTECTTVKDVP----KCITNNHMQDNLLDN--------TYLPKDVNRNFRSYEQVDVK------ 196  
PbR45    ------QSIKDS-----------ISDKSSKETVCPLESDINNNNN------NSTNVINYQNCDQKN-------ASSISNSCINKYKQLNSDNNN-NIGNC 202  
PyR45    ------QNIKDN-----------ISDKSSKETVCPFENDNNNNNNT-----NSTNVVNYQNCDKKN-------ESSISTSCINEYKQLNNDNN------- 195  

                 310       320       330       340       350       360       370       380       390       400         
         ....|....|....|....|....|....|....|....|....|....|....|....|....|....|....|....|....|....|....|....|
PgR45    YSYNVEENNKSIYILDNKKKKIETFLDYNSQNIMSIVNDDEKSYNNR------------------NNHKNIS---------------------DNTKDCL 302  
PkR45    GNRNGAKDSEPFCMGTGNSSIPSKDNAYYTNAYVHGKNDNSSEVVPYNHADSVVCEKEGVPTNPYSHGKDNQMEVTNYCIGGTVANGGGNLHDGDGKERL 387  
PvR45    GHKNGEKESNPFCMTTGKFSKQGEGSTDYNNAYVHGREDNSREVAS-------------------CHQTD-----------------------GE-KECL 301  
PrR45-2  --DNNSLLHTTIGCENKNKSYVNFPFNTVENKNIHIMMEEEKYRDHN----------------KVHHEID-------------------------DNGYI 253  
PbR45    NGNNKSVYNENINLEKSEIKNIEKNKSFDYCNDLHKNDQTSKELK--------------------YYETNH-------------------------EKLV 257  
PyR45    ----KNVYNENINLEKNEIKDIEKNKSFDYCNDLHNNDQTSKELK--------------------YYETNP-------------------------EKLV 246  

                 410       420       430       440       450       460       470       480       490       500         
         ....|....|....|....|....|....|....|....|....|....|....|....|....|....|....|....|....|....|....|....|
PgR45    KKNSSKLSCKKDNTINLTGNCYYDYHKIDNDPNFESSNALKLGNGKIEEYTN-----NINEIYGKKEDFRSMLS-LDIFNEMENINDSTTECNS-MKDIS 395  
PkR45    ANYPDMLMGNKWSDNVRGNGFHGGGHDIQDEDKCSTEDVIYMGMETSGSRGNQYERDSTDCLYGKKDDLKAIAYHQDIFNDIENMNDSTTECSSSVKDIP 487  
PvR45    ANCPDALMGGKWNEGLHVGGHLYADHHPQDEDKCSTEDVVYMGMQTSGSKAQQCEGESKNSPYVKKEDLKTIACQQDIFNDTENANDSTTECSSSVKDIP 401  
PrR45-2  INSRDDVVNNKDSFYKSLSQQIYIDKN-GSKYNVDISSDLFEKKCNINDKN-------MKNNYNENNDILRGAGQNDMPNNMYNISQKGFHLNQ--EGIN 343  
PbR45    YLEKNNLIDNNCNDIEYINDTTVDYISLKNLSQYSLNESLFKENNSN-DISN-----CNQFDHIINVNNSNSISNNDFFDNSHVKDSSSTKYSS-LKNNS 350  
PyR45    YLEKNNLIDNNCNDIEYINDTTVDYVSLKNLSQYSLNESLFRENNSN-DINN-----CKQFDHIINVNNSNSISNNDVFDNSHIKDSSLTKYSN-LKNNS 339  

                 510       520       530       540       550       560       570       580       590       600         
         ....|....|....|....|....|....|....|....|....|....|....|....|....|....|....|....|....|....|....|....|
PgR45    KFLVNYN--TSEKKLDKNYYINVPETPVSLKEINIQDKNSSS--ENISRKMSEMVLKSENKNGTPSNYDLDSIENNVPVINEDNFEIK----------KN 481  
PkR45    QFVANHSGVYSNRVIDKEGIVITSGHFSSYTDANNLEFKNSPSTKSFHLEKEKQ-LIYDNRGNIPVVSNGPNYVEE--EGEKNVFSEL-----VGGEPNK 579  
PvR45    QLVTNHPGVYANRVGEKEVVSITSGYFASYTDGKTFECKNSSAAKGPHSEKEKESIICDDLGFGPVVSNAANNGEAKVEGEKNAFAEAAQIAEVGGEPSN 501  
PrR45-2  DMLRNQN----NIAFQNNQYNGYIKDEQNIMN-NLCERIEP--------------------SYMDSMSKRDSEGTKGTVHKNNINRN------------N 406  
PbR45    QFYNKLTNNEDSAPFHEKKMKEKIDSNHASGNRNTYEIYK---------------------NEIGVSKNSNDVALNKCINVQGKFDDN-----------C 418  
PyR45    QFYDKLTNNGDSIAFHGKKMKEQIDSNNASDN-NTYEVYKSGAYKSETYKNETY-KNETYKNEIDTSKSSNNVSLNKCINVQENYDN------------C 425  

                 610       620       630       640       650       660       670       680       690       700         
         ....|....|....|....|....|....|....|....|....|....|....|....|....|....|....|....|....|....|....|....|
PgR45    ETIQQCFPN---KSIYMENDTHKCNEEFINDISDEEYTINKKEVKKDNYD------LSRNSFKVGSDIPNEIYLKKKSFNFNKKTDSIK----------- 561  
PkR45    GGMGVYLAGGYNNEALIPPGRNNNLLEKKCVMSEKKVQGGPISMEGSTPVGDMTSSTNGKGGSRNYNASNRDSVHGEGHIPNEALYK------------- 666  
PvR45    GAVGRYLAAGHTNKALCAPGVSDAAVEKKCVVSEKRGQGGPISIQVSPPT----SGTNINNGSRNFNSSKRDSLHGEARSPNEALYKKKSLYPGKEVDSM 597  
PrR45-2  NNHNGNINK---NNNNIHGNRNSNNIHGNRNSNNIHGNRNSNNIHSNRNS--------NN-THSNRNSNNSNRNNYNSFVVPGNMNSSN----------- 483  
PbR45    GERNKYIAN---DNKLNQDKINNDYKKYIDNNKSNICD-GSKNMGHHGES---DKWYSNKMNENNYIIEENDYQNNKNLHTNKEMNISK----------- 500  
PyR45    EERNEYIGN---DNKLNQDKINNGYKKYNDNNKSNIYGXGSKNMRHHGES---DKWYSNKMNSNNYIIEENDYHNNKNLHINKEMNISK----------- 508  

                 710       720       730       740       750       760       770       780       790       800         
         ....|....|....|....|....|....|....|....|....|....|....|....|....|....|....|....|....|....|....|....|
PgR45    -KKNDKTLTKSQIYESTKN-----------KNEESVNNLGKKKKKN----FFDLVSKR---------R---IESTVIPQ--DNSKDNVYLDEYFHNTIPS 631  
PkR45    -KKNETTLIKGQLPDYSRNEKNDESLEEYFKSTIPENILRNIKYENPVSTFNDIVSNNTHYDLTSELRKSNPTNTYYGSGRSGDMGGNKNGETMNNSGAA 765  
PvR45    KKKNETTLRKSQLMEYPQNE-NEESLEEYFKSTIPENILRNIKYENPSNTYNDMLSNNGHYDLTSELRNSNPTSTYYASGRGRDMGEKKSAEVVNNSGVA 696  
PrR45-2  --NNDNNDNNDNNYD--------KSLDEYFRNNLPEDIIKNIKYENPSNTYNDLLNNR---------------DYDLIS----------KIKENPVYYAS 548  
PbR45    -KKNDTTLKKSGIYEYRKYE-KNKCLEGYYHNEHSEINLRNVKCKNSSSGYNEIINNN--------NRNNNINSQYMIPSSQIGCNKNNKKKENMVYYEP 590  
PyR45    -KKNDTTLKKSGIYEYRKYE-KNKCLEGYHHNEHSEISLRNVKCENSASGYNEMVSNN--------NRNXNINSQYMIPPSQIGCNKNNKKKENVIYYES 598  

                 810       820       830       840       850       860       870       880       890       900         
         ....|....|....|....|....|....|....|....|....|....|....|....|....|....|....|....|....|....|....|....|
PgR45    DIMKNVKYENS-------------------------SNTYNEILNKMNYNLSSELNNNSIYISERNEEKNKKNRDTRDLNYIQNEKIGTRKEMQTHK--- 703  
PkR45    VASMGIRSRGA-------------VAEGAAVAGVPAIGGRDAYHVQADKGVDTREDSQMARRQNNGSYNYMGNNNYHGSGYNGNGYNGNSYNGNSYNGNG 852  
PvR45    SPGMNLRSRAAGGAASAAVAPGAAVATAAPVAGGTTVGGRDAYHVQADKGVDAREDPQAARKQNNGGYSYMG-SGYMGSGYNGGGYTGSGYNASGYNASG 795  
PrR45-2  EKEKKRMKN------------------------------KDMKQPKYVSNEKLDLDEHTHIYKNNNEDNNNN------------------NNNNSMN--- 597  
PbR45    RRNIDKMGG-------------------------RAEDMNSRMVTKINENIDSKEANENAVSYKDAHRSNIN--SIINCNNMNQNYHNKCNN-NYYN--- 659  
PyR45    RRNIDKMGG-------------------------RAGDMNSRMITKINENIDSKEANENAVSYKDAHRSNRN--SIINCNHINQNYHNNCNNSNYYN--- 668  

                 910       920       930       940       950       960       970       980       990       1000        
         ....|....|....|....|....|....|....|....|....|....|....|....|....|....|....|....|....|....|....|....|
PgR45    ----------------NDEGTKKIMCNELEANYTPGKR-YLLSVKKNEVIKDQKSQMLLISKQKIKKIWNKFKNNSSKEQNP-TTYDNKKYPFPNSD--- 782  
PkR45    YNGNGYN---------GMVNRSTRVCNEPESNYASGKRYYMLPPKKCDSVKDQKNQMLMISKQKIKKIWNKFKSGASKDQNLIPTFENEQNLFPNMDVLH 943  
PvR45    YNASGYNGYYSVEKDPGMATRNTRVCNEPESNYASGKRYYLLPPKKCDAVKEQKNQMLMISKQKIKKIWNKFKNGASKEQNLIPTFENEHNLFPNMEVLH 895  
PrR45-2  -----------------RDTTIISKCDNHEMHYGQGNSYILPTCQRNDLLKDDRNPMLSISKQKIKNIWSKFKNNTCKEHNVNRISESKNNNYVHKMEIM 680  
PbR45    -----------------PITENDIIHNEIDYNNIQGKKYYLASPKKNDGTKYSKNQMLMISKQKIKKMWNKFKNGS-KDQNISSTIEDENNMFPNAVEDC 741  
PyR45    -----------------SITENEIIHNEIDYNNIQGKRYYLPPSKKNDGTKYSKNQMLMISKQKIKKMWNKFKNGSPKDQNIGSTLDDENNMFPNVIEDC 751  

                 1010      1020      1030      1040      1050      1060      1070      1080      1090      1100        
         ....|....|....|....|....|....|....|....|....|....|....|....|....|....|....|....|....|....|....|....|
PgR45    -----------------------------------------------------------------LSTQKFEVCNYNS---------------------F 796  
PkR45    ASKMQGQ-GLSQMGSQMGTQIGGTQMNAQINAHGTPQMQAQLGVGIHPQVASPLH----TQLAGHVHSDFYGVSGGFQTCNQGGGGV-IGVGGGSLMSPF 1037 
PvR45    GQKVHAQIGGTQMNAPMNASLN-APMNAQINAS----LNAQLGVPMHPQVTPPLHGQLPGQLPGQMHSEFYGVSGPFQTCSQGAVGIGAGVGAGPLMSPF 990  
PrR45-2  NIPKKGLANEYYNYPYDNN----------------------------------------NNNNNNNNNNNNNNNNNNNSNSIINNN------------NN 728  
PbR45    N----------------------------------------------------------MQQKVQIYNDHYETNNDNHSN------------------GY 765  
PyR45    N----------------------------------------------------------IQQKVQIYNDHYETNNGNHSNN-----------------VY 776  

                 1110      1120      
         ....|....|....|....|....|..
PgR45    KDCINKPFN-----NINNMEKQIKSKC 818  
PkR45    KECISKPIR----RGSNVIEKQIKSKC 1060 
PvR45    KECINKPIR----RGSNVIEKQIKAKC 1013 
PrR45-2  KECINKPIIYMNHINNNNMDKQMRPKC 755  
PbR45    KDCVNKPIKWV--KNNN-IEKPIQSKC 789  
PyR45    KDCVNKPIKWV--KNNNNIEKPIQSKC 801  
